# Endorhizosphere of indigenous succulent halophytes: a valuable resource of plant growth promoting bacteria

**DOI:** 10.1186/s40793-023-00477-x

**Published:** 2023-03-18

**Authors:** Milica Dragojević, Nada Stankovic, Lidija Djokic, Vera Raičević, Jelena Jovičić-Petrović

**Affiliations:** 1 Faculty of Agriculture, University of Belgrade, Nemanjina 6, Zemun, 11080 Serbia; 2 Institute of Molecular Genetics and Genetic Engineering, University of Belgrade, Vojvode Stepe 444a, Belgrade, Serbia

**Keywords:** Endophytes, Halotolerance, Microbiota, PGP traits, Succulent halophytes

## Abstract

The adaptability of halophytes to increased soil salinity is related to complex rhizosphere interactions. In this study, an integrative approach, combining culture-independent and culture-dependent techniques was used to analyze the bacterial communities in the endorizosphere of indigenous succulent halophytes *Salicornia europaea*, *Suaeda maritima*, and *Camphorosma annua* from the natural salt marshes of Slano Kopovo (Serbia). The 16 S rDNA analyses gave, for the first time, an insight into the composition of the endophytic bacterial communities of *S. maritima* and *C. annua*. We have found that the composition of endophyte microbiomes in the same habitat is to some extent influenced by plant species. A cultivable portion of the halophyte microbiota was tested at different NaCl concentrations for the set of plant growth promoting (PGP) traits. Through the mining of indigenous halotolerant endophytes, we obtained a collection representing a core endophyte microbiome conferring desirable PGP traits. The majority (65%) of the selected strains belonged to the common halotolerant/halophilic genera *Halomonas*, *Kushneria*, and *Halobacillus*, with representatives exhibiting multiple PGP traits, and retaining beneficial traits in conditions of the increased salinity. The results suggest that the root endosphere of halophytes is a valuable source of PGP bacteria supporting plant growth and fitness in salt-affected soils.

**Supplementary Information:**

The online version contains supplementary material available at 10.1186/s40793-023-00477-x.

## Introduction

Halophytes, which make up about 1% of the world’s flora species [20], developed various strategies to cope with salinity stress and to survive and reproduce across different saline environments. The increased accumulation of salts has adverse effects on soil physicochemical and biological properties, disturbing the ecological balance and affecting the growth and diversity of organisms living on and in the soil [45]. Acting through osmotic stress, ion toxicity, nutrient imbalance, or their combination [60] the increased salinity causes many harmful effects on plants. Its influence on basic physiological processes such as germination, vegetative growth, and reproduction [60] results in decreased agricultural production and low economic returns. However, it has been shown that the adaptability of plants to different habitats and their survival, particularly under stressful conditions, are influenced by their associated microbial communities, termed as plant microbiota [55]. Due to the co-evolution with the plant in the same hostile environment, the microbiota of halophytes is adapted to the salty habitat and is considered to be the key factor for understanding the adaptation of plants to the saline habitat [16].

A vital part of plant microbiota is represented by Plant Growth Promoting (PGP) bacteria which inhabit the rhizosphere, phyllosphere, and endosphere and support plant growth and health through multifarious PGP traits  [34].

Endophyte microorganisms found in plant root tissues represent a subset of the root microbiota [22] and spend at least parts of their life cycle inside the plant without apparent harm to the host [36]. Whether they originate from the surrounding environment such as bulk soil and rhizosphere or being transmitted via seed [36], endophytic bacteria play a crucial role in plant development, growth, and diversification [29]. Unlike halophilic microorganisms which require salt for growth, halotolerant microorganisms can grow in the absence of NaCl as well as in a wide range of NaCl concentrations, up to 25% in the case of bacteria [69, 16].

As a part of the halophyte microbiota, halotolerant plant growth-promoting endophytic bacteria (HPGPE) [76] can contribute to the plant host growth, productivity, and fitness under abiotic and biotic stress [21] through phytostimulation, biofertilization, and biocontrol [22]. The important way of phytostimulation involves 1-aminocyclopropane-1-carboxylate (ACC) deaminase activity, which prevents ethylene from reaching growth-inhibitory concentrations in the plant and represents one of the key bacterial physiological traits that facilitate plant growth (especially) under stress conditions [23].

Exploring the diversity of endophytic bacterial communities of various indigenous halophytes is an initial step toward a better understanding of plant-microbial-soil relations under adverse salinity conditions.

In this study, we aim to explore the endophytic bacterial community from three species of succulent halophytes, *Salicornia europaea* L., *Suaeda maritima* (L.) Dumort., and *Camphorosma annua* Pall., indigenous to natural salt marshes of Slano Kopovo in Vojvodina, a center of biodiversity and representative example of fragile salt marsh habitats that have almost disappeared in this part of Europe.

The aim of the present study was to assess the biodiversity of endophytic root microbiota communities using an integrative approach combining culture-dependent and culture-independent techniques. The culture-dependent techniques enabled the characterization of individual microbiota members, providing insight into the possible ways by which HPGPE support host plant under natural conditions of increased salinity. On the other hand, a profound understanding of plant-microbe interactions requires a wider, culture-independent, analysis of the plant root endorhizosphere microbiota.

## Methods

### Site description and plant material sampling

The plant material was collected from naturally formed saline lands at Special Nature Reserve „Slano Kopovo“, located in Vojvodina, Serbia. In 2004, Slano Kopovo was included in the list of Ramsar areas and became an internationally recognized important plant area (IPA).

The following three species of characteristic halophyte succulents were selected: *Salicornia europaea*, *Suaeda maritima*, and *Camphorosma annua*, all members of the same family *Chenopodiaceae*. Three individual and spatially distant plants were randomly selected for each species. The top 0.5–1 cm of soil covered with salt crust was removed and the whole plants, including the surrounding soil, were dug out with a spade. The plant material was transported in sterile plastic bags and the roots and rhizosphere samples were processed within 24 h.

### The collection and separation of root endosphere and rhizosphere

A common rhizosphere sample of three collected individuals was made for each plant species. The roots were gently shaken to remove loosely adherent soil. Rhizosphere soil was separated from the root endosphere according to Schlaeppi et al. [57] with modifications. Briefly, the collected roots were cut into 3 cm long segments, starting 0.5 cm from the root base. Root segments were placed in an Erlenmeyer flask containing 100 ml of phosphate buffered saline (PBS) and shaken at 180 rpm for 20 min (GFL 3005, Germany) for initial washing. The root washing in PBS was repeated two more times under the same conditions. After short drying on sterile Whatman filter paper, the roots were macerated in sterile saline using a sterile mortar and pestle.

One fraction of the macerated root samples was preserved using DNA/RNA Shield reagent (Zymo Research, USA) (1:10) for microbiota 16 S rDNA analysis while the rest was used for isolation of bacteria.

### 16 S rDNA analysis of endorhizosphere microbiota

The samples were processed and analyzed with the ZymoBIOMICS® Targeted Sequencing Service for Microbiome Analysis (Zymo Research, Irvine, CA) using their standard pipeline. In short, DNA was extracted with the ZymoBIOMICS® DNA Miniprep Kit (Zymo Research, Irvine, CA). Bacterial 16 S ribosomal RNA gene targeted sequencing was performed using the *Quick*-16 S™ NGS Library Prep Kit (Zymo Research, Irvine, CA) with Zymo Research custom-designed primers. The final library was sequenced on Illumina® MiSeq™ with a v3 reagent kit (600 cycles). The sequencing was performed with > 10% PhiX spike-in. Subsequently, the unique amplicon sequences were inferred from raw reads using the DADA2 pipeline [7]. Chimeric sequences were also removed with the DADA2 pipeline. Chimeras and singletons were filtered from the dataset before analysis and operational taxonomic units (OTUs) were clustered at 94 and 97% identity, roughly corresponding to genus and species level, respectively. Taxonomy assignment was performed using Uclust from Qiime v.1.9.1 [8] with the Zymo Research 16 S Database internally designed and curated, as a reference database. If applicable, a taxonomy that had significant abundance among different groups was identified by LEfSe [58] using default settings. The community structure of three plants microbiota was compared by principal coordinate analysis (PCoA) created using the matrix of the paired-wise distance between samples calculated by the Bray-Curtis (BC) dissimilarity.

### Isolation of halotolerant endophytic bacteria

For isolation of halotolerant endophytes, ten-fold serial dilutions of macerated root tissue in sterile saline were prepared. The resulting suspensions were plated in triplicate on Nutrient agar, NA, (Torlak, Serbia) supplemented with NaCl (5, 10 and 15% (w/v)) and filter-sterilized root tissue extract (1% (w/v)). The plates were incubated at 30 °C until the colonies appeared. Morphologically different colonies were picked and the characteristics of selected isolates (colony morphology, cellular morphology, Gram staining, motility, and endospore forming) were determined using standard procedures. The obtained isolates were stored at − 80 °C in 25% glycerol.

### Isolation of ACC deaminase producing endophytic bacteria

ACC deaminase producing root endophytes were isolated following the procedures described by Penrose and Glick [49]. To enrich the population of pseudomonads and similar Gram-negative bacteria in the sample, 1 g of the macerated root was incubated in PAF media [49] for 48 h. A 1 ml aliquots of enriched cultures were transferred to 50 ml sterile DF salts minimal medium [13] with (NH_4_)_2_SO_4_ as a nitrogen source and incubated on the orbital platform shaker at 200 rpm and 30 °C. After 24 h, 1 ml of culture was transferred to a 50 ml DF minimal medium supplemented with 3 mM ACC as the sole nitrogen source and incubated overnight. Subsequently, the cultures were diluted, plated on a solid DF medium with 3 mM final ACC (Sigma–Aldrich, USA), and incubated for 72 h at 30 °C.

The colonies that grew on media with ACC as the sole nitrogen source were transferred and maintained on NA plates for further analysis (see section "Quantification of ACC-deaminase activity").

### Screening for salt tolerance

All isolates were screened for salt tolerance by inoculating NA plates supplemented with different concentrations of NaCl (0, 3, 5, 7, 10, 12, 15, 18, 20, and 25%) with the overnight culture of endophytes and incubating on 30 °C. After 48 h, the plates were observed for growth.

### PGP activity screening

All PGP assays were performed in the presence of 3% and 7% NaCl (w/v) to assess if isolates will retain their PGP properties in elevated salinity.

#### Quantification of ACC-deaminase activity

The confirmation and quantification of ACC utilizing ability was performed using a colorimetric assay based on the ninhydrin reaction [37] using 96-well PCR plates.

After overnight incubation in Luria broth, LB (tryptone 10 g/L, NaCl 10 g/L, yeast extract 5 g/L) and centrifugation at 8000 xg for 5 min, the cell pellet was washed twice, resuspended in 2 ml of fresh DF medium supplemented with 3 mM ACC as sole nitrogen source, and incubated at 28 °C/200 rpm/24 h. A sample of uninoculated DF-ACC medium was incubated as a control. After incubation, 1 ml of each culture was centrifuged at 8000×*g* for 5 min and the supernatant was diluted ten-fold. The 60 µl of diluted supernatant was mixed with 120 µl of ninhydrin reagent in a PCR plate and incubated in a boiling water bath for 30 min. The analysis of each sample was performed in triplicate. The change of Ruheman’s purple color in samples in comparison to the control DF-ACC medium indicated ACC utilizing bacteria. The ACC consumptions were quantified by transferring reaction solutions into a flat-bottom 96-well microtiter plate and measuring absorbance at 570 nm (TECAN, Switzerland). The obtained absorbance values were compared to a standard curve created by using the increasing concentrations of ACC (5–500 µM/L).

#### Indole acetic acid (IAA) production

The IAA production was assessed by the colorimetric assay [47]. Overnight bacterial cultures were transferred to 9 ml of minimal salt media (M9 minimal salts 200 ml/L,1 M MgSO_4_ 2 ml/L, 1 M CaCl_2_ 0.1 ml/L, 20% D-glucose 20 ml/L; M9 minimal salts: Na_2_HPO_4_ × 7H_2_O 64 g/L, KH_2_PO_4_ 15 g/L, NaCl 2,5 g/L, NH_4_Cl g/L), supplemented with 100 µg/ml of L-tryptophan (Sigma Aldrich, USA) and incubated for 72 h at 30 °C and 150 rpm. The supernatant obtained by centrifugation at 6000×*g*/15 min was mixed with Salkowski reagent in a ratio of 1:2. The absorbance at 540 nm (T70 UV / VIS Spectrometer, PG Instruments Ltd) of developed pink color after the 25 min incubation indicated the production of IAA. The concentrations (µg/ml) were determined from a standard curve created using IAA standards (Sigma-Aldrich, USA) in concentrations ranging between 1 and 100 µg/ml.

#### Ammonia production

The isolates were grown in 5 ml Peptone water (Torlak, Serbia) for 72 h at 30 °C. After incubation, 0.5 ml of Nessler’s reagent (Alfapanonia, Serbia) was added to each tube. The color change to bright yellow or brown indicated a positive reaction for ammonia production [9].

#### Siderophore production

Siderophore production was determined according to the modified Chrome Azurol S (CAS) assay described in Lakshaman et al. [35]. The solid medium supplemented with CAS (Fluka, USA) was spot inoculated with individual colonies and incubated at 30 °C for 5 days. A positive reaction for the siderophore production was monitored by the development of a yellow-orange halo around the inoculation site.

#### Exopolysaccharide (EPS) production

The EPS production was determined according to Paulo et al. [48]. Five µl of overnight bacterial cultures were inoculated on the sterile filter paper discs (5 mm Ø) placed on solid medium for EPS production [27] and incubated for 48 h at 30 °C. The appearance of slimy substances around the discs indicated the production of EPS, while the formation of precipitates in tubes after mixing the slime with 2 ml of 96% ethanol confirmed the reaction.

#### Biofilm-forming ability

The ability of the isolates to form biofilms was examined by colorimetric crystal violet assay on 96-well microtiter plates according to Stepanović et al. [63] with modifications. After overnight growth of bacteria in LB at 30 °C, the media were removed and wells were washed twice with PBS to remove the non-adherent cells. Biofilms were fixed with methanol, air dried, and stained with 0.4% crystal violet for 15 min. To dissolve the dye, stained biofilms were incubated for 20 min in 33% acetic acid. Absorbance was measured at 630 nm in a microtiter plate reader (TECAN, Switzerland). The sterile medium was used as a negative control. The isolates were classified into the following categories by comparing obtained average OD values to OD_c_ value defined as three standard deviations (SD) above the average OD of the negative control according to Stepanović et al. ([63]):

OD (isolate) ≤ ODc = biofilm non producing.

ODc ≤ OD (isolate) ≤ 2ODc = weak biofilm-producing.

2ODc ≤ OD (isolate) ≤ 4ODc = moderate biofilm-producing.

4ODc ≤ OD (isolate) = strong biofilm-producing.

#### P, Zn, and K solubilization assays

National Botanical Research Institute’s phosphate (NBRIP) medium with Ca_3_(PO_4_)_2_ was used to test the ability of bacterial isolates to dissolve inorganic phosphates according to Nautiyal [43]. The spot inoculated NBRIP medium plates were incubated for 14 days at 30 °C.

The ability of the isolates to solubilize inorganic sources of Zn was determined on a mineral medium supplemented with 1% ZnO [24] that was incubated for 72 h at 30 °C.

Aleksandrov medium containing K-Al silicates as K-bearing minerals supplemented with brome thymol blue was used for screening the K solubilization potential of isolates [53]. The inoculated plates were incubated for 72 h at 30 °C.

All solubilization assays were performed in triplicate by spotting overnight bacterial culture on test-specific solid media. The appearance of clear zones around colonies indicated the ability of tested bacteria to solubilize inorganic sources of P or Zn, while the appearance of a yellow halo around the colonies was considered as a positive reaction for K solubilization.

The solubilizing ability of isolates was expressed via the solubilization index (Si) and calculated as follows: (colony diameter + halo zone diameter)/colony diameter.

### 16 S rRNA gene sequencing of selected HPGPE

The selected isolates were identified by 16 S rRNA gene sequencing. Genomic DNAs were isolated using Zymo Research Soil Microbe DNA Mini Prep Kit (Zymo Research, USA), according to the manufacturer’s instructions. Genes coding for 16 S rRNAs were amplified with bacteria-specific primers 27f and 1492r [38]. Generated PCR products were sequenced using BigDye® Terminator v3.1 Sequencing Kit (Applied Biosystems) using same primers and run on Applied Biosystems 3130 Genetic Analyser (Applied Biosystems, Foster City, USA). Sequences were analyzed and assembled using the SeqMan tool from DNASTAR Software (Lasergene). BlastN program [4] was used to search for similar sequences in the GenBank database services provided by the NCBI.

The partial 16 S rRNA gene sequences of selected strains were deposited in the GenBank under accession numbers given in Table 1.

## Results

### Microbiota composition analysis

**Fig. 1:**
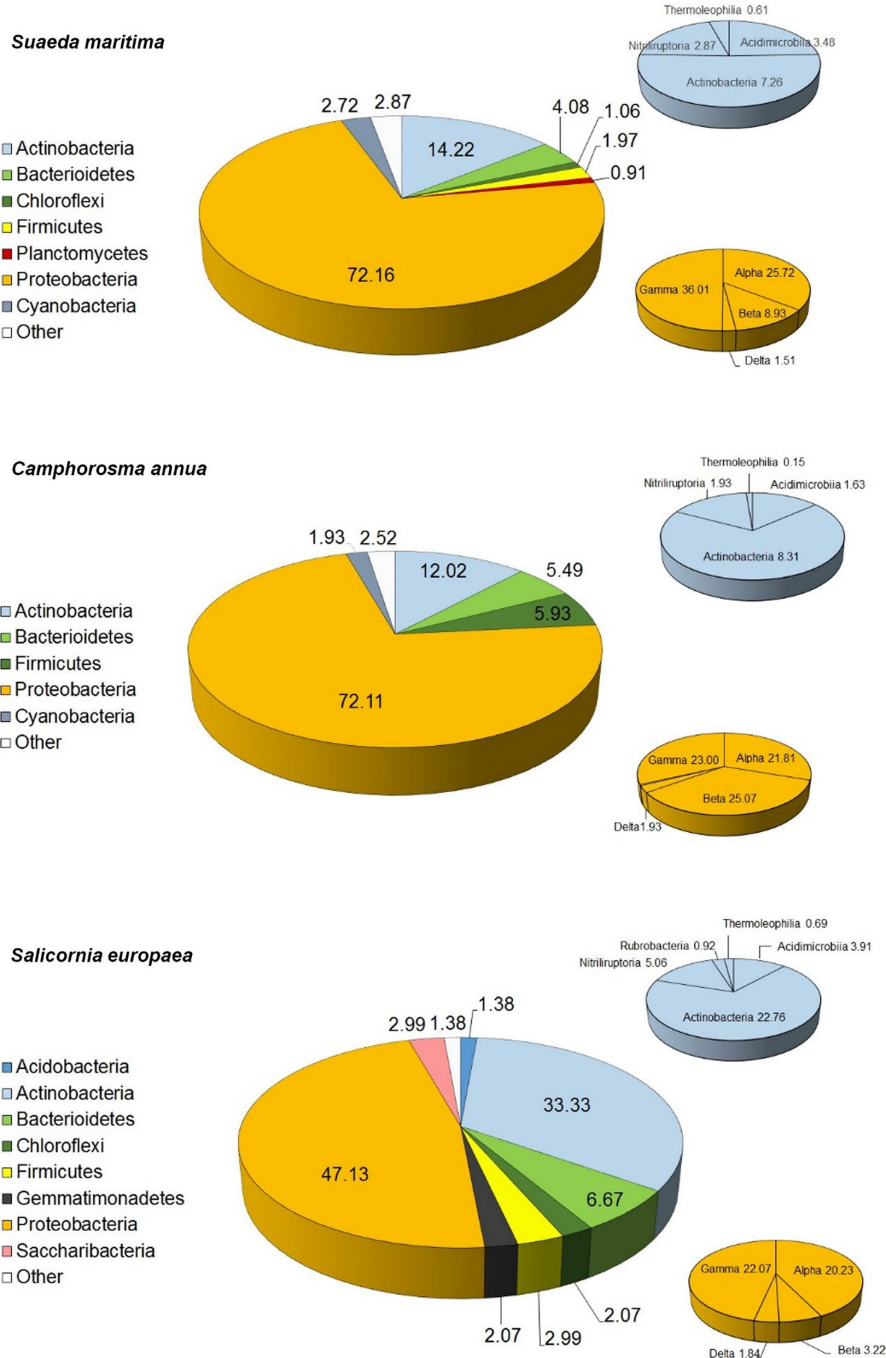
Distribution of phyla in *S. maritima*, *C. annua*, and *S. europaea*, and distribution of classes within two major phyla (Actinobacteria and Proteobacteria). Distribution of most abundant classes in Proteobacteria (Alpha-, Beta-, Gamma- and Deltaproteobacteria) are shown in yellow pies, while the most abundant Actinobacteria (Actinobacteria, Acidimicrobiia, Nitriliruptoria) are shown in blues pies. Most of Bacterioidetes make classes Cytophagia (3.33% in *S. maritima*, 4.30% in *C. annua*, and 3.22% in *S. europaea*) and Flavobacteria (in *S. europaea* 3.22%), in Firmicutes class Bacilli makes 5.19% in *C. annua* and 2.53% in *S. europaea*

The most abundant bacterial phyla in all three plants were Proteobacteria (72% in *S. maritima* and *C. annua*; 47% in *S. europaea*) and Actinobacteria (14% in *S. maritima*, 12% in *C. annua*, 33% in *S. europaea*), comprising roughly 80% of each microbiota. Other prominent phyla detected were Bacteroidetes and Cyanobacteria in *S. maritima*, and Firmicutes and Bacteroidetes in both *C. annua* and *S. europaea* with the addition of Saccharibacteria to the most abundant phyla in *S. europaea* (Fig. 1).

**Fig. 2:**
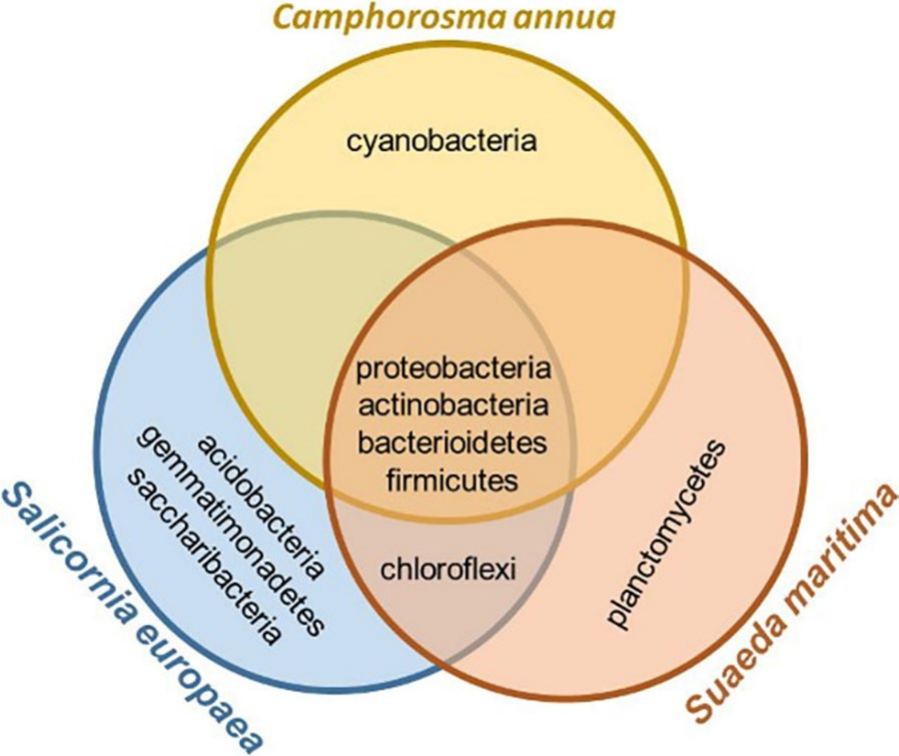
Complexity of phyla distribution in three halophyte plants

*C. annua* showed the lowest diversity of subdominant phyla (> 1% of relative abundance), counting only three, while complexity of the composition of subdominant phyla was higher in *S. maritima* (5 phyla) and *S. europaea* (6 phyla) as shown in Fig. 2.

Examination of the relative abundances of phyla, classes (Fig. 1), and orders (Additional file 1: Table SM1) primarily revealed differences between *S. maritima* and *C. annua* on one side, and *S. europaea* on the other side.

At the family level, by far the most abundant family in *S. maritima* and *C. annua* were Pseudomonadaceae (16.64% and 12.02%, respectively) followed by Rhodocyclaceae (6.96% and 19.29%). In *S. europaea*, there was no single prominent family, but four families with percentile abundances between 6 and 8% (Promicromonosporaceae 8.05%, Rhodobacteraceae 6.44%, Halomonadaceae 7.82%, and Pseudomonadaceae 5.98%). A more detailed distribution of families in all three plant endorhizosphere is given in Additional file 1: Table SM2.

The results on alpha diversity showed variation in species abundance between *S. maritima*, *C. annua*, and *S. europaea* (374, 400, and 264 unique reads per 16,230 reads, respectively). PCoA plots of beta diversity of *S. maritima* and *C. annua* locate closely indicating that these two plants mostly contain the same bacterial genera and have similar microbial composition profiles (Additional file 1: Figure SM3). BC dissimilarity between *S. europaea* compared to *S. maritima* and *C. annua* was below 0.25, suggesting a somewhat different microbial composition profile.

**Fig. 3:**
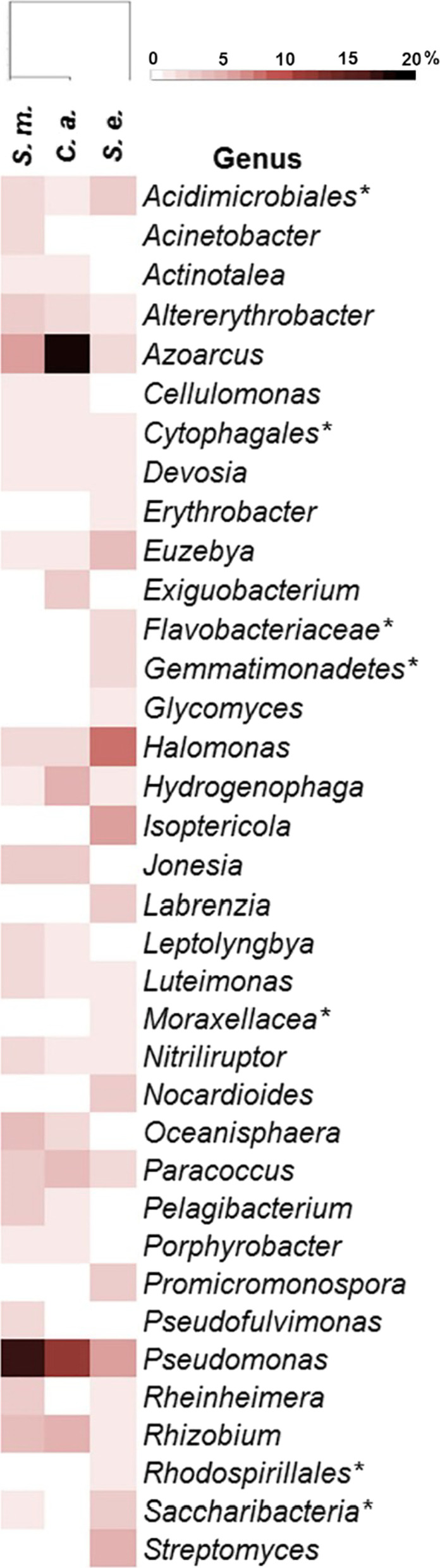
Biodiversity of genera between three halophyte plants. *S.m*., *Suaeda maritima*, *C.a*., *Camphorosma annua*, *S.e*., *Salicornia europaea.* (*) signifies rare bacteria where taxonomy could be determined only up to family level

An analysis of the distribution of the most abundant OTUs between the three plants revealed that only 19.09% of genera were present in all three plants, where 62.75%, 60.32%, and 25.56% were shared between *S. maritima* and *C. annua*, *C. annua* and *S. europaea*, and *S. maritima* and *S. europaea*, respectively. Sample clustering showed that *S. maritima* and *C. annua* shared most of the abundant genera (genera having over 1% abundance), differing only by the presence of one unidentified genus from the phylum Saccharibacteria (Additional file 1: Table SM4, Fig. 3).

At the species level, there were several representatives of certain genera that stood out both in percentage and in number. Genus *Azoarcus* was represented with 6 species in *S. maritima*, 8 species in *C. annua*, and 3 species in *S. Europaea*. Notedly, genus *Azoarcus* was most prominent in *C. annua* with abundance of 17.66%. *Pseudomonas* was the other genus with marked diversity in all three plants. The microbiota of *S. maritima* harbored 11 different *Pseudomonas* species and the genus was most prominent with 16.64% abundance, while *C. annua* and *S. europaea* harbored 10 and 8 *Pseudomonas* species respectively. Notable was the presence of species with high identity to *Pseudomonas pseudoalcaligenes* that made 5.45% in *S. maritima* and 6.82% in *C. annua*. Apart from members of *Azoarcus* and *Pseudomonas* genera, *S. maritima* and *C. annua* shared distinct presence of several other bacterial species belonging to genera *Hydrogenophaga, Altererythrobacter*, *Paracoccus*, *Jonesia*, *Pelagibacterium*, *Rhizobium*, *Luteimonas aestuarii*, and *Oceanisphaera*, all present in percentage between 1 and 4% except *Oceanisphaera* sp.56,188 (4.24% in *S. maritima*). While the presence of *Streptomyces* genus was neglectable in *S. maritima* and *C. annua*, in *S. europaea* it contributed with 4.83% counting 3 species. The table of species detected in all three plants is given in the supplementary data (Additional file 1: Table SM5).

### Collection of endophyte isolates

A total of 32 endophytes were isolated from the root tissues of *S. maritima, C. annua*, and *S. europaea.* Of these, 20 isolates were selected based on their ability to exhibit at least two PGP traits at 3% NaCl. The majority of halotolerant isolates had bright, orange, or yellow colored colonies (87%) and 80% were Gram-negative and rod-shaped. On the other hand, 20% of the isolates were Gram-positive and rod-shaped, except one strain of cocci.

Five selected Gram-negative isolates (14A1, 14A2, 15A1, 15A2, 16A1) were able to grow on DF agar containing ACC, which indicated their ACC deaminase activity. Isolates 14A1 and 14A2 originated from *S. maritima* were coccobacilli, with whitish and opaque colonies on NA. Isolates 15A1 and 15A2 from *C. annua* and 16A1 from *S. europaea* were short, rod- shaped with creamy and translucent colonies on NA.

### Molecular identification of isolates

Taxonomic positions of isolates were determined based on the sequence of the 16 S rRNA genes. Isolated endophytes were identified as belonging to six different genera: *Acinetobacter* (2 strains), *Kushneria* (4 strains), *Pseudomonas* (3 strains), *Halomonas* (7 strains), *Halobacillus* (2 strains), *Planococcus* (1 strain), and *Klebsiella* (1 strain). The percentage of 16 S rRNA gene sequence similarities (98.93–100%) of these isolates to the closest type strains is presented in Table 1.

**Table 1:** Morphological characteristics, salt tolerance, and taxonomic identity of root endophytes associated with succulent halophytes

Plant of origin	Isolate	Colony pigment	Gram stain/cell morphology	Max NaCl	Genus	Closest species (% similarity)	Accession No.
*S. maritima*	14A2	W/Op	–/Cocobacilli	3%	*Acinetobacter*	*Acinetobacter calcoaceticus* (100)	OL625645
	14A5	W/Op	–/Cocobacilli	3%	*Klebsiella*	*Klebsiella aerogenes* (99.93)	OL625646
	14.2	W/Op	–/Cocobacilli	18%	*Acinetobacter*	*Acinetobacter calcoaceticus* (99,79)	OK668372
	14KX1	O/T	–/Rods	18%	*Kushneria*	*Kushneria pakistanensis* (99,17)	OL625647
	14KX2	Y/Op	–/Rods	18%	*Halomonas*	*Halomonas meridiana/ Halomonas songnenensis* (100)	OL625648
*C. annua*	15K13	Y/Op	–/Rods	16%	*Halomonas*	*Halomonas songnenensis* (100)	OL627342
	15.2	Y/Op	–/Rods	12%	*Halomonas*	*Halomonas songnenensis* (100)	OL627338
	15K2	R–O/Op	+/Cocci	16%	*Planococcus*	*Planococcus rifietoensis* (99,88)	OL627341
	15A1	C/T	+/Rods	3%	*Pseudomonas*	*Pseudomonas putida* (99,71)	OL627339
	15A2	C/T	–/Rods	3%	*Pseudomonas*	*Pseudomonas fluorescens* (99,78)	OL627340
*S. europaea*	16A1	C/T	–/Rods	3%	*Pseudomonas*	*Pseudomonas putida* (99,93)	OL657066
	16.1	O/T	–/Rods	16%	*Kushneria*	*Kushneria indalinina* (98,93)	OL657063
	16.2	O/T	–/Rods	18%	*Kushneria*	*Kushneria indalinina* (98,93)	OL657064
	16.4	W/Op	+/Rods	25%	*Halobacillus*	*Halobacillus andaensis* (99,93)	OL657065
	16K2	Y/Op	–/Rods	16%	*Halomonas*	*Halomonas songnenensis* (99,93)	OL657067
	16K5	Y/Op	–/Rods	18%	*Halomonas*	*Halomonas songnenensis* (99,86)	OL657068
	16K7	Y/Op	–/Rods	18%	*Halomonas*	*Halomonas songnenensis* (99,86)	OL657069
	16KX1	O/T	–/Rods	18%	*Kushneria*	*Kushneria indalinina* (99,86)	OL657070
	16KX2	Y/Op	+/Rods	25%	*Halobacillus*	*Halobacillus massiliensis* ( 99,45)	OL657072
	16KX3	O/Op	–/Rods	18%	*Halomonas*	*Halomonas songnenensis* (99,87)	OL657071

W, white; Y, yellow; O, orange; R, red; Op, opaque; T, translucent; C, creamy; (–), Gram stain negative; (+), Gram stain positive

### Salt tolerance of isolated bacteria

The isolated endophytes display variable growth at different salt concentrations (Table 1). All tested strains were able to grow on NA without additional NaCl. The members of the *Halobacillus* genus showed the capacity to grow at the highest salinity level (25% NaCl). *Kushneria* and *Halomonas* strains were able to grow in a wide range of NaCl concentrations up to 18%, while *Pseudomonas*, *Acinetobacter*, and *Klebsiella* strains isolated as ACC deaminase producers, couldn’t grow at salt concentrations higher than 3%.

### PGP activities of isolates

The PGP activities of isolated endophytes were tested at different concentrations of NaCl (0, 3, and 7% NaCl (w/v)). Isolates that were able to grow only in up to 3% NaCl were tested for PGP traits only in 0 and 3% NaCl. The obtained results for the PGP potential of isolates were summarized in Table 2. In the absence of NaCl, all isolates exhibited at least three, while 20% of isolates exhibited 7 of 8 tested PGP traits.

Ninhydrin test revealed that five strains isolated on DF media with ACC as sole nitrogen source could utilize and decrease the initial concentration of 3 mM ACC in DF media during 16 h incubation. After growing in media supplemented with L-tryptophan, 9 bacteria were positive for IAA production. The amount of produced IAA ranged from 2.10 to 4.62 µg ml^-1^. The strain *Halomonas* sp. 14KX2 was found to produce the highest amounts of IAA (4.62 and 4.00 µg ml in 0% and 3% NaCl, respectively). Another *Halomonas* strain, 16K2, could produce IAA across the entire range of NaCl concentrations with the maximal production at 7% NaCl. With increasing salt concentration, 56% of IAA producing strains lost that capability.

The qualitative assessment of ammonia production showed that 90% of tested bacteria produced ammonia in the absence of NaCl and retained the production in the presence of 3% NaCl. In media amended with 7% NaCl, 22% of isolates (14.2, 15K2, 16.4, and 16KX2) lost the ability to produce ammonia.

In CAS medium without NaCl, 75% of strains formed the orange halos surrounding colonies indicating the siderophore production. Three *Kushneria* (14KX1, 16.1, and 16KX1) and two *Halomonas* (14KX2 and 15K13) strains retained the ability of siderophore production at each of the tested salt concentrations.

EPS were produced by 95% of tested bacteria in media without NaCl and 47% of them maintained that feature in the presence of various NaCl concentrations.

Out of all tested bacteria, 35% showed ability for K, P, or Zn solubilization. Five ACC deaminase producers (14A2, 14A5, 15A1, 15A2, and 16A1) showed the potential to solubilize potassium, phosphorus, and zinc from inorganic sources in the media without and with 3% NaCl. The solubilization of nutrients on 7% NaCl could be established only for one halotolerant isolate, *Kuschneria* 16.1, which could solubilize phosphorus in the presence of every tested concentration of NaCl. The nutrient solubilization ability was found to be inversely proportional to salt concentration. Among nutrient solubilizing strains *Klebsiella* 14A5 exhibited the highest Si values for K (8.64 and 5.12 at 0% and 3% NaCl, respectively), P (4.53 and 3.29 at 0% and 3% NaCl, respectively), and Zn (5.05 and 5.55 at 0% and 3% NaCl, respectively).

The biofilm-forming ability of selected strains was affected by the increase of NaCl concentration (Table 2). All of the tested isolates showed biofilm-forming ability in the absence of NaCl, which was classified as weak (55% of strains) and moderate (40%). At 3% NaCl, 50% of isolates were classified as weak, 30% as moderate biofilm producers, while *Acinetobacter* 14A5 lost its biofilm-forming ability. *Halomonas* 16K2 showed the highest OD_630_ value of 0.203 at 3% NaCl and retained moderate biofilm production at 7% NaCl concentration. At 7% NaCl, 26.6% of isolates lost the ability to form biofilms while others retained biofilm production (60% as weak and 13.4% as moderate).

**Table 2:** Plant growth-promoting ability of endophytes at different salt concentrations

Isolate	ACC consumption^a^	Production																Solubilization index (SI) (cm)										
		IAA (µg ml^−1^)			Ammonia			Siderophore			EPS			Biofilm^b^				Potassium				Phosphate				Zinc		
		NaCl concentration % (w/v)																										
		0	3	7	0	3	7	0	3	7	0	3	7	0	3	7		0	3	7		0	3	7		0	3	7
14A2	2.71	−	−	/	+	+	/	+	−	/	+	+	/	M	W	/		7.39	2.58	/		3.47	2.96	/		4.27	4.57	/
14A5	2.77	−	−	/	+	+	/	+	+	/	+	+	/	W	N	/		8.64	5.12	/		4.53	3.29	/		5.05	5.55	/
14.2	−	−	5.0	−	+	+	−	+	+	−	+	−	−	W	W	W		2.29	−	−		−	−	−		−	−	−
14KX1	−	−	−	−	+	+	+	+	+	+	+	+	+	W	N	N		−	−	−		−	−	−		−	−	−
14KX2	−	4.62	4.00	−	+	+	+	+	+	+	+	+	+	M	M	W		−	−	−		−	−	−		−	−	−
15K13	−	−	−	−	+	+	+	+	+	+	+	+	+	M	W	W		−	−	−		−	−	−		−	−	−
15.2	−	4.13	−	−	+	+	+	−	−	−	+	+	+	M	N	W		−	−	−		−	−	−		−	−	−
15K2	−	4.00	−	−	+	+	−	+	+	−	+	−	−	W	W	N		−	−	−		−	−	−		−	−	−
15A1	2.75	−	−	/	+	+	/	+	+	/	+	+	/	M	W	/		5.33	2.72	/		2.87	−	/		4.27	2.60	/
15A2	2.80	−	−	/	+	+	/	+	+	/	+	+	/	W	N	/		5.23	2.55	/		2.92	2.40	/		3.10	2.76	/
16A1	2.73	−	−	/	+	+	/	+	+	/	−	−	/	W	W	/		5.86	3.13	/		2.95	−	/		4.03	3.14	/
16.1	−	−	−	−	−	−	−	+	+	+	+	+	+	M	M	N		−	−	−		2.60	2.51	2.29		−	−	−
16.2	−	4.13	−	−	−	−	−	−	−	−	+	+	+	W	W	W		−	−	−		−	−	−		−	−	−
16.4	−	4.10	−	−	+	+	−	+	+	−	+	−	−	W	M	W		−	−	−		−	−	−		−	−	−
16K2	−	2.10	2.40	2.93	+	+	+	−	−	−	+	+	−	W	M	M		−	−	−		−	−	−		−	−	−
16K5	−	3.46	−	−	+	+	+	−	−	−	+	+	+	W	W	W		−	−	−		−	−	−		−	−	−
16K7	−	−	−	−	+	+	+	−	−	−	+	+	+	M	M	M		−	−	−		−	−	−		−	−	−
16KX1	−	−	−	−	+	+	+	+	+	+	+	+	+	M	M	W		−	−	−		−	−	−		−	−	−
16KX2	−	−	−	−	+	+	−	+	+	−	−	+	−	N	W	N		−	−	−		−	−	−		−	−	−
16KX3	−	4.10	2.85	−	+	+	+	+	+	−	−	+	−	W	W	W		−	−	−		−	−	−		−	−	−

+, positive, −, negative, /, unable to grow

^a^The ACC consumption value of each isolate was calculated by subtracting the value of ACC concentration after incubation from the initial concentration of 3 mM in DF medium

^b^Biofilm: N, non producing; W, weak producing; M, moderate producing; S, strong producing

## Discussion

The seal of Slano Kopovo is characterized by its specific vegetation, related to the flora of salt marshes and consisting of diverse halophytes among which succulents stand out. *S. europaea*, *S. maritima* and *C. annua* grow on extremely saline and periodically flooded solonchak to highly saline solonchak and solonetz under moderate humidity conditions [39]. They represent a part of genuine halophyte plant cover which has almost disappeared from most of the Pannonian area [66].

There is a limited number of studies on the structure of endophytic communities associated with halophytes in different natural saline habitats. Only a few studies describe endophytic communities associated with *Salicornia* genus [18], especially *Salicornia europaea* [21, 64, 72, 73], and even fewer describe the endophytic microbiota associated with representatives of *Suaeda* genus [71, 75]. There are no reported data on endophyte microbiota communities associated with *Suaeda maritima* and *Camphorosma* genus in the current literature.

The shore of the Slano Kopovo salt lake was the location where all samples were collected, so the diversity of halotolerant halophyte associated endophytic bacteria was not expected to depend on the physico-chemical properties of soil but rather on specificity of the plant host. Our results showed that all three plants shared substantial portion of microbiota (50% of the most abundant genera in all three plants) but that the distribution of the major bacterial phyla was almost identical for *S. maritima* and *C. annua* and differed for *S. europaea*. *C. annua* showed the lowest diversity of taxa, whereas compositional complexity was higher in *S. maritima* and *S. europaea*. This structural community complexity pattern of three plant endophytic microbiota (*C. annua* < *S. maritima* < *S. europaea*) was repeated down all taxonomic levels up to genus level.

The majority of abundant genera and their most prominent species in the microbiota were, unsurprisingly, previously described as marine or halotolerant organisms or were described as yellow-pigmented bacteria. The majority of isolates obtained as a cultivable portion of the halophyte microbiota had yellow to orange pigmented colonies, indicating potential carotenoid production. Production of pigments with significant antioxidant and antimicrobial activity which help bacteria to cope with stress conditions, photo-oxidation, and free radicals is common at high salinity (Fariq et al. [17]). For the less common genera in analyzed microbiota, there is no literature data on some specific PGP trait or interaction with plants. However, some of them are present in a fairly high percentage (5.52% of bacteria with high identity to *Isoptericola halotolerans* in *S. europaea*, 4.24% of members of genus *Ocenisphera* in *S. maritima*, or 4.75% of *Hydrogenophaga* in *C. annua* root endophytic microbiota) suggesting the adaptive advantage they bring to the plant host.

The other portion of the most abundant genera in three plants endophytic microbiota consists of genera whose PGP traits have been well documented in the literature (Table 3). *Streptomyces* genus is renowned for its secondary metabolites that are in human use as antibiotics and antimicotics. This considerable potential was obviously harnessed by plants as well, to serve biocontrol purposes [10]. *Streptomyces* contribute to plant wellbeing in various manners possessing a “full package” of PGP traits (IAA, ACC, siderophore production, and even the ability to fix N_2_) [70]. *Rhizobium* is a genus found in all three plants and is known for its nitrogen fixation ability and, recently, for various other PGP traits [68]. *Halomonas* genus was expected to be present in the halophyte host environment (in *S. europaea* this genus makes for 7.82% of endophyte microbiota) and under saline stress produces exopolysaccharides [42], but also have IAA and siderophore producing ability [19]. Some species of the genus show phosphate solubilization, ammonia and ACC deaminase production, and nitrogen fixation abilities [40]. Our endophyte isolation strategy allowed us to cultivate and characterize seven *Halomonas* sp. Isolates, six of which are closely related (> 99.86% similarity) to *Halomonas songnenensis*, a moderately halophilic bacterium from saline and alkaline soils [31]. *Halomonas* sp. were previously noted as a root endophyte of *S. brachiata* [30]. *Azoarcus* genus is present in all three plants microbiota, but in *C. annua* it accounts for an astonishing 17.66%. This is another genus containing nitrogen fixating and IAA producing species [54] that seems to be devoid of plant pathogenic traits but possesses genes suggesting a biocontrol role [33]. Finally, the most prominent genus in all three plants endophytic microbiota, making between 6 and 17%, was the genus *Pseudomonas*. There are numerous *Pseudomonas* strains that are characterized as PGP, as well as plant pathogens. The strains that promote plant growth are able to synthetize IAA, cytokines, iron-chelating molecules, and antimicrobial peptides, and have the ability of nitrogen fixation [50, 74]. *Pseudomonas pseudoalcaligenes*, the most abundant species in the *S. maritima* and *C. annua* microbiota (5.45% and 6.82%, respectively), is proved to have various PGP traits [32] as well as the ability to tolerate high salinity. These PGP abilities are present in 3 *Pseudomonas* strains isolated from *C. annua* and *S. europaea*.

**Table 3:** PGP traits of most common and abundant genera in three plants’ endophyte microbiota

Genus or species	Abundance			PGP trait(s)
	*S. maritima* (%)	*C. annua* (%)	*S. europaea* (%)	
*Streptomyces*	0.15	0.15	**4.83**	IAA, ACC deaminase, siderophore production, biocontrol [70]
*Rhizobium* *Leguminosarum* *Rosettiformans-vitis*	**4.39**	**4.90**	1.15	N2 fixation, siderophore, IAA, ammonia, ACC deaminase production, phosphate solubilization [68]
*Halomonas* *Chromatireducens* *Desiderata* *Nitritophilus* *Songnenensis*	1.97	1.78	**7.82**	Exopolysaccharide [42], IAA, siderophore [19] production, P solubilization, NH_3_ and ACC deaminase production, N_2_ fixation [40]
*Azoarccus*	**6.05**	**17.66**	2.07	N2 fixation, IAA production [54]
*Pseudomonas* *Pseudoalcaligenes* *Anguilliseptica-guineae-peli* *Composti* *Indoloxydans-oleovorans* *Kuykendallii* *Mendocina* *Stutzeri* *Xanthomarina-zhaodongensis*	**16.64**	**12.02**	**5.98**	IAA, cytokinines, siderophore, N_2_ fixation, biocontrol [50, 74, 32]

The most abundant genera for given plant species are presented in bold lettering

Yamamoto et al. [73] showed that endophyte diversity and the community structure associated with each halophyte differed between two nearby natural saline sites. Comparing *S. europaea* endophytes from different and geographically distant habitats showed that none of the common genera in the mentioned study of *S. europaea* endophyte seemed to be even present in *S. europaea* from Slano Kopovo. Although strikingly different genera form the endophytic microbiome of various studied *Suaeda* plants, depending on physico-chemical composition of the soil and soil bacteria available for recruiting, the potential PGP traits these microbiota are conveying have been persistent.

The diversity of halotolerant endophytes associated with halophyte roots suggests that different halophyte adopt the same ecological strategies to combat salinity stress by attracting microbiota with specific metabolic traits. These traits can be further harnessed from the cultivable portion of the endophytic microbiome to improve agricultural plants’ growth and salt tolerance. In *Suaeda salsa* microbiota contributing to salt stress acclimatization, nutrient solubilization, and competitive root colonization were detected forming a potential core microbiome [75] capable to support plant wellbeing. We expect in further work to be able to form such a potential core microbiome based on cultivable halotolerant endophytic bacteria that we described in this study.

### A cultivable fraction of HPGPE

The identification by 16 S rRNA gene sequencing revealed the distribution of selected cultivable endophytes into seven genera belonging to two phyla, Proteobacteria (85%) and Firmicutes (15%).

The majority of cultivable endophytes detected in this study belong to the common halotolerant/halophilic genera *Halomonas*, *Kushneria*, and *Halobacillus* (Table 1). The genera *Halomonas* and *Halobacillus* include halophiles that are adaptable to salinity changes and thus are able to grow in a wide range of salt concentrations [28]. The genus *Kushneria*, along with *Halomonas*, is an important member of the family Halomonadaceae, a taxonomic group within the Gammaproteobacteria that are typical of hypersaline environments [40]. Taxonomic identification of the obtained isolates showed that *Halomonas* was the predominant genus within the halotolerant root-associated bacteria of all three halophytes. Genera *Halomonas*, *Kushneria*, and *Halobacillus* reported in this study as endophyte, were isolated from the rhizosphere of *Salicornia* [40], while *Kushneria* is found exclusively as endophytic in leaves of *Salicornia* [41] and halophyte *Arthrocnemum macrostachyum* [44]. In contrast to *Halomonas*, a genus abundant in all three plant microbiota, and *Halobacillus* that is a rare genus in *S. europaea* (0.23%), from whose microbiome two isolates were obtained, *Kushneria* was below the detection level in *S. maritima* and *C. annua*, and present only 0.0009% among *S. europaea* microbiota. We presume that the cultivation conditions that we applied were favorable for the isolation of this rare genus.

*Acinetobacter* is a genus found across various habitats, often in harsh conditions like hypersaline environments [11] and it is also commonly found as an endophyte [3]. The species with high similarity to *A. haemoliticus* was among the highly abundant species among endophytes of *S. maritima* (2.27%), however, the two isolated *Acinetobacter* sp. from the same plant had the highest similarity to *A. calcoaceticus* (99.79% and 100% similarity) which was not detected in 16 S rDNA analysis. Another cultivated bacteria that was below the level of detection was *Klebsiella* isolated on DF media from a root of *S. maritima. Klebsiella* isolate was 99.93% similar to *K. aerogenes*, reported in the leaves of *Suaeda salsa* [59].

Due to nutritional and metabolic versatility, *Pseudomonas* species inhabit diverse habitats demonstrating different lifestyles [3], including endophytic [29]. Gram-positive endophyte *Planococcus* were isolated from *C. annua* roots and identified as *P. rifietoensis* (99.88%). *P. rifietoensis* was reported as an endophyte associated with *S. europaea* in Zhao et al. [76].

### Halotolerance of selected HPGPE

In terms of salt tolerance, the isolates belonging to the genera *Halomonas*, *Kushneria*, *Halobacilus*, and *Planococcus* showed the ability to grow in a broad range of salt concentrations (12–25%). Bearing in mind that saline soils are characterized by significant heterogeneity in salt distribution and a wide range of salt concentrations surrounding one single plant [6], a level of halotolerance is an important criterion to select strains for a potential application. Bacterial strategy involving the synthesis or uptake of compatible organic solutes to maintain osmotic cell potential, makes them more flexible and adaptable to changing salinity [28]. This type of adaptation is widespread among members of the Halomonadaceae family [28].

In addition to developing mechanisms for their survival under stress, HPGP increase plant host tolerance to salinity stress (Shrivarstava and Kumar 2015) through mechanisms that act in an integrated manner to mitigate hostile environmental impacts. In the current study, tested endophytes exhibited multiple PGP features even under conditions of elevated NaCl concentration, suggesting their role in host growth promotion and contribution to abiotic stress tolerance.

### PGP potential of halotolerant endophytes

The phytohormone activity has a key role in plant adaptations to diverse stresses. Production of IAA, the most prevalent auxin in plants, is an important feature of HPGPE that affects plant growth. 45% of the isolates distributed among five genera showed the ability to produce IAA (Table 2). All of them produced IAA in the absence of NaCl, except *Acinetobacter* 14.2 which produced IAA only at 3% NaCl. *Planococcus*, *Kuchneria*, *Halobacillus*, and some *Halomonas* strains lost the ability to produce IAA at increased salt concentrations. Upadhyay et al. [67] reported that the level of IAA produced by isolates decreased with increasing NaCl concentration. In this study, *Halomonas* 16K2, isolated from *S.europaea* (*Halomonas songnenensis* 99.93% similarity) stands out because of the positive correlation between IAA production and substrate salt concentration.

Boosting the growth of roots and stems, exogenous IAA improves the exploitation of nutrients from saline soil and can contribute to leaf growth which is important for plant productivity under saline conditions [12, 16]. Inoculation of wheat with halotolerant IAA producing bacteria, isolated from highly saline habitats, including *Halomonas* sp., resulted in improved fitness of wheat on salt-affected soil [65] indicating a bacterial role in augmenting plant resistance to salt stress.

Lowering ethylene levels is the most prominent bacterial PGP feature in alleviating harmful effects of stress on plants [15, 16].

Isolates that showed positive ACC deaminase activity in this study belonged to the genera *Acinetobacter*, *Klebsiella*, and *Pseudomonas* (Table 2). ACC deaminase activity among *Pseudomonas* species is widely observed [2, 49], while *Acinetobacter* ACC deaminase activity along with *Pseudomonas* was reported by Govindasamy et al. [26]. Multiple-PGP *Klebsiella* sp. SBP-8 role in induced systemic tolerance in wheat under salt stress has been reported [62]. Siddike et al. [61], noted that halotolerant bacteria expressing IAA and ACC deaminase are more effective in stimulating plant cell elongation and dry weight in canola than bacteria which were sole ACC deaminase producers. None of the isolates in this study showed both PGP abilities, but the obtained collection brings the possibility for the design of effective consortia for potential application.

Under saline conditions, plant growth and productivity are reduced because of nutrient imbalance [16]. By increasing the availability of nutrients to plants, endophytes can enhance directly their growth and fitness.

Ammonia production is a common PGP trait that directly supports plant growth via nitrogen supply [46]. The majority of isolates showed the ability of ammonia production at increased salt concentrations. Two *Kushneria* strains could not produce ammonia, but this was not a genus-specific pattern as the third strain tested positive at any salt concentration. Ammonia production appears to be a frequent trait of *Halomonas*, as all isolates were positive and retained this ability with increasing salt concentrations.

Growing on salty soils, plants are simultaneously affected by salinity and a limited amount of bioavailable Fe, which is a common problem for calcareous and saline-sodic soils around the world [1]. Halotolerant PGPE, especially those associated with halophytes, produce Fe siderophores which represent small, soluble complexes that can be easily absorbed by plants [15, 45]. In this study, the siderophore production ability varied among members of the same genus (Table 2). Among *Kushneria* and *Halomonas* were the strains that produced siderophore in presence of increased salt concentration indicating a contribution to the availability of Fe to host plants. All *Pseudomonas* strains were positive for siderophore production.

Phosphorus is an essential plant nutrient required for all main processes in plant physiology. Phosphate solubilization activity is a PGP feature of halotolerant bacteria associated with halophytes [5, 77]. Besides strains with ACC deaminase activity, only *Kushneria* 16.1, showed and retained phosphate solubilization ability at every tested salt concentration. The high phosphate solubilization ability of halotolerant *Kushneria* isolated from saltern sediments was reported in Zhu et al. [78]. In the present study, *Acinetobacter* 14A2, *Klebsiella*, and *Pseudomonas fluorescens* expressed phosphate solubilization ability at their maximum salt tolerant concentration of 3% NaCl (Table 2).

In most of the soil K is in a form inaccessible to plants, but PGP bacteria can solubilize K-bearing minerals and release potassium that plants can uptake [14]. In this study, ACC-positive strains demonstrated remarkable potential for solubilization of K, P, and Zn. ACC-positive strains in this study possess multiple PGP traits (produce ammonia, siderophore, EPS, and biofilm) indicating that ACC deaminase positive bacteria are among the most potent PGP microbes and unequivocally support the growth and survival of their host.

As a co-factor of many enzymes, zinc is an important micronutrient that plays a vital role in various metabolic processes in plants, and its deficiency adversely affects the growth and development of plants [24, 45]. In terms of zinc solubilization ability, *Klebsiella* and *Acinetobacter* 14A2 (Table 2) stood out by the size of the halo zone and the positive correlation with the salt concentration. In the case of *Pseudomonas* strains, their zinc solubilization ability slightly decreased with increasing salt concentrations. Zinc solubilization ability is recorded for *Acinetobacter* strain isolated from rice roots [24], Gontia-Mishra et al. [25] reported the zinc solubilization ability of ACC-positive *Klebsiella pneumonie* and *Pseudomonas aeruginosa* which exhibited multiple PGP traits such as P and K solubilization.

The production of EPS and biofilm formation are important PGP features under the conditions of salt-imposed stress. Due to the ability to bind cations, bacterial EPS restrict Na^+^ available for plant uptake [67], and shield the root from high salt concentrations (Ruppel et al. [56]). EPS are important components that support biofilm expansion and stabilize its structure, but an abundance of EPS is not an indication for biofilm formation [51]. As highly hydrating substances with cementing features, bacterial EPS plays a vital role in the formation and stabilization of soil aggregates thus improving soil structure (Otlewska et al. [45]). *Halomonas meridiana* 14KX2 and *Halomonas songnenensis* 16K2 stood out for their ability to produce biofilm and EPS at different salinity levels. *Halomonas meridiana* PAa6 showed maximal biofilm production at 1 M NaCl (~ 5.5% NaCl) and was more effective in biofilm formation, EPS production, and root colonization under saline and non-saline conditions in comparison to *Kushneria indalinina* and *Halomonas aquamarina* [52]. Similarly, in our study *Kushneria indalinina* strains 16.1 and 16.2, produced EPS in the presence of increased salt concentration but were less effective in biofilm formation in comparison to *Halomonas meridian*a 14KX2. Halotolerant *Halomonas variabilis* and *Planococcus riftoensis* strains showed a positive correlation between EPS production and biofilm-forming and increasing salinity. These strains had a positive influence on the formation of aggregates in the soil and on plant roots [52]. Generally, in our study increasing salt concentration induced reduction but not complete loss of the ability of tested isolates for EPS production and biofilm-forming (Table 2). The results indicate that bacteria that stayed positive for the tested features could potentially develop communities under salt stress and colonize plant roots and soil particles, which is vital for potential application in salt-affected soils. Among the representatives of the genus *Halomonas*, which dominated among cultivated bacteria and showed multiple PGP properties, 14KKS2 and 16K2 *Halomonas songinensis* stood out because they retained PGP traits under conditions of increased salinity.

## Conclusion

Our results suggest that halophytes’ root endosphere is a significant source for the selection of bacteria with a potential to improve plant fitness under saline stress. Therefore, besides salinity being a key environmental factor, plant species are also determinative for the endophyte community composition. The relative abundances of phyla, classes, and orders primarily revealed differences between *S. maritima* and *C. annua* on one side, and *S. europaea* on the other side. Within the presented work, we obtained a cultivable part of halophyte microbiomes exhibiting all PGP traits important for nutrition, growth stimulation, and stress resistance of plants. Thus, the obtained collection provides a solid basis for the formulation of consortium containing core endophyte microbiome conferring all desirable traits. The results also highlight the potential of using habitat-adapted, indigenous endophytic bacteria to enhance the growth and ameliorate abiotic stress damage of host plants growing in special habitats.

## Supplementary Information

**Additional file 1**. Supplementary Information.

## Data Availability

The data generated during this study are included in this article and its Additional file 1.

## References

[1] Abbas H, Patel RM, Parekh VB (2018). Culturable endophytic bacteria from halotolerant *Salicornia brachata* L.: isolation and plant growth promoting traits. IJAM.

[2] Ali S, Charles TC, Glick BR (2014). Amelioration of high salinity stress damage by plant growth-promoting bacterial endophytes that contain ACC deaminase. Plant Physiol Biochem.

[3] Alibrandi P, Schnell S, Perotto S, Cardinale M (2020). Diversity and structure of the endophytic bacterial communities associated with three terrestrial orchid species as revealed by 16S rRNA gene metabarcoding. Front Microbiol.

[4] Altschul SF, Gish W, Miller W, Myers EW, Lipman DJ (1990). Basic local alignment search tool. J Mol Biol.

[5] Aslam F, Ali B (2018). Halotolerant Bacterial Diversity Associated with *Suaeda fruticosa* (L.) Forssk. Improved growth of maize under salinity stress. Agronomy.

[6] Bazihizina N, Barrett-Lennard E, Colmer T (2012). Plant growth and physiology under heterogeneous salinity. Plant Soil.

[7] Callahan BJ, McMurdie PJ, Rosen MJ, Han AW, Johnson AJ, Holmes SP (2016). DADA2: high resolution sample inference from Illumina amplicon data. Nat Methods.

[8] Caporaso JG, Kuczynski J, Stombaugh J, Bittinger K, Bushman FD, Costello EK, Fierer N, Peña AG, Goodrich JK, Gordon JI (2010). QIIME allows analysis of high-throughput community sequencing data. Nat Methods.

[9] Cappuccino JG, Sherman N (1996). Microbiology-A Laboratory Manual.

[10] Colombo EM, Kunova A, Pizzatti C, Saracchi M, Cortesi P, Pasquali M (2019). Selection of an endophytic *Streptomyces* sp. strain DEF09 from wheat roots as a biocontrol agent against *Fusarium graminearum*. Front Microbiol.

[11] Dahal RH, Chaudhary DK, Kim J (2017). *Acinetobacter halotolerans* sp. nov., a novel halotolerant, alkalitolerant, and hydrocarbon degrading bacterium, isolated from soil. Arch Microbiol.

[12] Dodd IC, Pérez-Alfocea F (2012). Microbial amelioration of crop salinity stress. J Exp Bot.

[13] Dworkin M, Foster JW (1958). Experiments with some microorganisms which utilize ethane and hydrogen. J Bacteriol.

[14] Etesami H, Emami S, Alikhani H (2017). Potassium solubilizing bacteria (KSB): mechanisms, promotion of plant growth, and future prospects: a review. J Soil Sci Plant Nutr.

[15] Etesami H, Beattie GA (2018). Mining halophytes for plant growth-promoting halotolerant bacteria to enhance the salinity tolerance of non-halophytic crops. Front Microbiol.

[16] Etesami H, Glick BR (2020). Halotolerant plant growth–promoting bacteria: prospects for alleviating salinity stress in plants. Environ Exp Bot.

[17] Fariq A, Yasmin A, Jamil M (2019). Production, characterization and antimicrobial activities of bio-pigments by *Aquisalibacillus elongatus* MB592, *Salinicoccus sesuvii* MB597, and *Halomonas aquamarina* MB598 isolated from Khewra Salt Range. Pakistan Extremophiles.

[18] Ferreira MJ, Cunha A, Figueiredo S, Faustino P, Patinha C, Silva H, Sierra-Garcia IN (2021). The root microbiome of *Salicornia ramosissima* as a seedbank for plant-growth promoting halotolerant bacteria. Appl Sci.

[19] Figueroa LO, Schwarz B, Richards AM (2015). Structural characterization of amphiphilic siderophores produced by a soda lake isolate, *Halomonas* sp. SL01, reveals cysteine-, phenylalanine- and proline-containing head groups. Extremophiles.

[20] Flowers TJ, Colmer TD (2008). Salinity tolerance in halophytes. New Phytol.

[21] Furtado BU, Gołębiewski M, Skorupa M, Hulisz P, Hrynkiewicz K (2019). Bacterial and fungal endophytic microbiomes of *Salicornia europaea*. Appl Environ Microbiol.

[22] Gaiero JR, McCall CA, Thompson KA, Day NJ, Best AS, Dunfield KE (2013). Inside the root microbiome: bacterial root endophytes and plant growth promotion. Am J Bot.

[23] Gamalero E, Glick BR (2015). Bacterial modulation of plant ethylene levels. Plant Physiol.

[24] Gandhi A, Muralidharan G (2016). Assessment of zinc solubilizing potentiality of *Acinetobacter* sp. isolated from rice rhizosphere. Euro J Soil Biol.

[25] Gontia-Mishra I, Sapre S, Tiwari S (2017). Zinc solubilizing bacteria from the rhizosphere of rice as prospective modulator of zinc biofortification in rice. Rhizosphere.

[26] Govindasamy V, Raina SK, George P, Kumar M, Rane J, Minhas PS, Vittal KPR (2017). Functional and phylogenetic diversity of cultivable rhizobacterial endophytes of sorghum [*Sorghum bicolor* (L.) Moench]. Antonie Van Leeuwenhoek.

[27] Guimarães DP, Costa FAA, Rodrigues MI, Maugeri F (1999). Optimization of dextran synthesis and acidic hydrolisis by surface response analysis. Braz J Chem Eng.

[28] Gunde-Cimerman N, Plemenitaš A, Oren A (2018). Strategies of adaptation of microorganisms of the three domains of life to high salt concentrations. FEMS Microbiol Rev.

[29] Hardoim PR, van Overbeek LS, Berg G, Pirttilä AM, Compant S, Campisano A, Döring M, Sessitsch A (2015). The hidden world within plants: ecological and evolutionary considerations for defining functioning of microbial endophytes. Microbiol Mol Biol Rev.

[30] Jha B, Gontia I, Hartmann A (2012). The roots of the halophyte *Salicornia brachiata* are a source of new halotolerant diazotrophic bacteria with plant growth-promoting potential. Plant Soil.

[31] Jiang J, Pan Y, Hu S, Zhang X, Hu B, Huang H, Hong S, Meng J, Li C, Wang K (2014). *Halomonas songnenensis* sp. nov., a moderately halophilic bacterium isolated from saline and alkaline soils. Int J Syst Evol Microb.

[32] Khan MMA, Duan J, Glick BR, Finnegan PM, Kabli SA, Al-Garni SMS (2021). Draft genome sequence of the plant growth-promoting bacterium *Pseudomonas pseudoalcaligenes* KB-10. Microbiol Resour Announc.

[33] Krause A, Ramakumar A, Bartels D, Battistoni F, Bekel T, Boch J, Böhm M, Friedrich F, Hurek T, Krause L (2006). Complete genome of the mutualistic, N2-fixing grass endophyte *Azoarcus* sp. strain BH72. Nat Biotechnol.

[34] Kumawat KC, Sharma P, Nagpal S, Gupta RK, Sirari A, Nair RM, Bindumadhava H, Singh S (2021). Dual microbial inoculation, a game changer? - bacterial biostimulants with multifunctional growth promoting traits to mitigate salinity stress in spring mungbean. Front Microbiol.

[35] Lakshmanan V, Shantharaj D, Li G, Seyfferth LA, Sherrieri AL, Bais HP (2015). A natural rice rhizospheric bacterium abates arsenic accumulation in rice (Oryza sativa L). Planta.

[36] Lata R, Chowdhury S, Gond SK, White JF (2018). Induction of abiotic stress tolerance in plants by endophytic microbes. Lett Appl Microbiol.

[37] Li Z, Chang S, Lin L, Li Y, An Q (2011). A colorimetric assay of 1-aminocyclopropane-1-carboxylate (ACC) based on ninhydrin reaction for rapid screening of bacteria containing ACC deaminase. Lett Appl Microbiol.

[38] Lane DJ, Stackebrandt E, Goodfellow M (1991). 16S/23S rRNA sequencing. Nucleic acid techniques in bacterial systematic.

[39] Luković MS. Vegetation of saline habitats of Serbia with an assessment of the sustainable use and conservation [dissertation]. Belgrade (BG): University of Belgrade, Serbia; 2019.

[40] Mapelli F, Marasco R, Rolli E, Barbato M, Cherif H, Guesmi A, Ouzari I, Daffonchio D, Borin S. Potential for plant growth promotion of rhizobacteria associated with *Salicornia* growing in Tunisian hypersaline soils. Biomed Res Int. 2013;248078. 10.1155/2013/248078 PMC3679824 23781499

[41] Mora-Ruiz Mdel R, Font-Verdera F, Díaz-Gil C, Urdiain M, Rodríguez-Valdecantos G, González B, Orfila A, Rosselló-Móra R (2015). Moderate halophilic bacteria colonizing the phylloplane of halophytes of the subfamily Salicornioideae (Amaranthaceae). Syst Appl Microbiol.

[42] Mukherjee P, Mitra A, Roy M (2019). *Halomonas* rhizobacteria of *Avicennia marina* of indian sundarbans promote rice growth under saline and heavy metal stresses through exopolysaccharide production. Front Microbiol.

[43] Nautiyal CS (1999). An efficient microbiological growth medium for screening phosphate solubilizing microorganisms. FEMS Microbiol Lett.

[44] Navarro-Torre S, Mateos-Naranjo E, Caviedes MA, Pajuelo E, Rodríguez-Llorente ID (2016). Isolation of plant-growth-promoting and metal-resistant cultivable bacteria from *Arthrocnemum macrostachyum* in the Odiel marshes with potential use in phytoremediation. Mar Pollut Bull.

[45] Otlewska A, Migliore M, Dybka-Stępień K, Manfredini A, Struszczyk-Świta K, Napoli R, Białkowska A, Canfora L, Pinzari F (2020). When salt meddles between plant, soil, and microorganisms. Front Plant Sci.

[46] Orhan F (2016). Alleviation of salt stress by halotolerant and halophilic plant growth-promoting bacteria in wheat (*Triticum aestivum*). Braz J Microbiol.

[47] Patten CL, Glick BR (2002). Role of *Pseudomonas putida* indole acetic acid in development of host plant root system. Appl Environ Microbiol.

[48] Paulo ME, Vasconcelos PM, Oliveira SI, Affe MH, Nascimento R, De Melo SI, De Abreu Roque MR, De Assis SA (2012). An alternative method for screening lactic acid bacteria for the production of exopolysaccharides with rapid confirmation. Food Sci Technol.

[49] Penrose MD, Glick RB (2003). Methods for isolating and characterizing ACC deaminase- containing plant growth-promoting rhizobacteria. Physiol Plant.

[50] Preston GM (2004). Plant perceptions of plant growth-promoting *Pseudomonas*. Philos Trans R Soc Lond B Biol Sci.

[51] Qurashi AW, Sabri AN (2012). Bacterial exopolysaccharide and biofilm formation stimulate chickpea growth and soil aggregation under salt stress. Braz J Microbiol.

[52] Qurashi AW, Sabri AN (2012). Biofilm formation in moderately halophilic bacteria is influenced by varying salinity levels. J Basic Microbiol.

[53] Rajawat MVS, Singh S, Tyagi SP, Saxena AK (2016). A modifed plate assay for rapid screening of potassium-solubilizing bacteria. Pedosphere.

[54] Reinhold B, Hurek T, Gillis M, Hoste B, Vancanneyt M, Kersters K, Ley J (1993). *Azoarcus* gen. nov., Nitrogen-fixing Proteobacteria associated with roots of Kallar Grass (*Leptochloa fusca* (L.) Kunth), and description of two species, *Azoarcus indigens* sp. nov. and *Azoarcus communis* sp. nov.. Int J Syst Evol Microb..

[55] Rodríguez-Llorente ID, Pajuelo E, Navarro-Torre S, Mesa-Marín J, Caviedes MA, Kumar M, Etesami H, Kumar V (2019). Bacterial endophytes from halophytes: how do they help plants to alleviate salt stress?. Saline soil-based agriculture by halotolerant microorganisms.

[56] Ruppel S, Franken P, Witzel K (2013). Properties of the halophyte microbiome and their implications for plant salt tolerance. Funct Plant Biol.

[57] Schlaeppi K, Domborowski N, Oter RG, van Ver Loren E, Schulze-Lefert P (2014). Quantitative divergence of the bacterial root microbiota in *Arabidopsis thaliana* relatives. Proc Natl Acad Sci U S A.

[58] Segata N, Izard J, Waldron L, Gevers D, Miropolsky L, Garrett WS, Huttenhower C (2011). Metagenomic biomarker discovery and explanation. Genome Biol.

[59] Shang N, Ding M, Dai M, Si H, Li S, Zhao G (2019). Biodegradation of malachite green by an endophytic bacterium Klebsiella aerogenes S27 involving a novel oxidoreductase. Appl Microbiol Biotechnol.

[60] Shrivastava P, Kumar R (2015). Soil salinity: a serious environmental issue and plant growth promoting bacteria as one of the tools for its alleviation. Saudi J Biol Sci.

[61] Siddikee MA, Chauhan PS, Anandham R, Han GH, Sa T (2010). Isolation, characterization and use for plant growth promotion under salt stress, of ACC deaminase-producing halotolerant bacteria derived from coastal soil. J Microbiol Biotechnol.

[62] Singh RP, Jha P, Jha PN (2015). The plant-growth-promoting bacterium *Klebsiella* sp. SBP-8 confers induced systemic tolerance in wheat (*Triticum aestivum*) under salt stress. J Plant Physiol.

[63] Stepanović S, Vuković D, Hola V, Di Bonaventura G, Djukiv S, Cirković I, Ruzicka F (2007). Quanitification of biofilm in microtiter plates: overview of testing conditions and practical recommendationsa for assessment of biofilm production by staphylococci. APMIS.

[64] Szymańska S, Borruso L, Brusetti L, Hulisz P, Furtado B, Hrynkiewicz K (2018). Bacterial microbiome of root-associated endophytes of Salicornia europaea in correspondence to different levels of salinity. Environ Sci Pollut Res Int.

[65] Tiwari S, Singh P, Tiwari R, Meena KK, Yandigeri M, Singh DP, Arora DK (2011). Salt-tolerant rhizobacteria-mediated induced tolerance in wheat (*Triticum aestivum*) and chemical diversity in rhizosphere enhance plant growth. Biol Fertil Soils.

[66] Tomić R, Mrkša M, Kovačević-Berleković B, Maksimović B, Dragosavac M, Imbronjev M. 2016. Nautical tourism sustainable resources of the municipality of Novi Bečej. *Экономика, экология и общество России в 21-м столетии* (EESR-2021), 198.

[67] Upadhyay SK, Singh JS, Singh DP (2011). Exopolysaccharide plant growth promoting Rhizobacteria under salinity condition. Pedosphere.

[68] Vejan P, Abdullah R, Khadiran T, Ismail S, Nasrulhaq Boyce A (2016). Role of Plant Growth promoting Rhizobacteria in Agricultural Sustainability—a review. Molecules.

[69] Ventosa A, Mellado E, Sánchez-Porro C, Márquez MC, Dion P, Nautiyal CS (2008). Halophilic and halotolerant micro-organisms from soils. Microbiology of Extreme Soils.

[70] Vurukonda SSKP, Giovanardi D, Stefani E (2018). Plant growth promoting and biocontrol activity of *Streptomyces* spp. as endophytes. Int J Mol Sci.

[71] Wang H, Narsing Rao MP, Gao Y, Li X, Gao R, Xie Y, Li Q, Li W (2021). Insights into the endophytic bacterial community comparison and their potential role in the dimorphic seeds of halophyte *Suaeda glauca*. BMC Microbiol.

[72] Yamamoto K, Shiwa Y, Ishige T, Sakamoto H, Tanaka K, Uchino M, Tanaka N, Oguri S, Saitoh H, Tsushima S (2018). Bacterial diversity associated with the rhizosphere and endosphere of two halophytes: *Glaux maritima* and *Salicornia europaea*. Front Microbiol.

[73] Yamamoto K, Matsutani M, Shiwa Y, Ishige T, Sakamoto H, Saitoh H, Tsushima S (2020). Comparative analysis of bacterial diversity and community structure in the rhizosphere and root endosphere of two halophytes, *Salicornia europaea* and *Glaux maritima*, collected from two brackish lakes in Japan. Microbes Environ.

[74] Yan Y, Yang J, Dou Y, Chen M, Ping S, Peng J, Lu W, Zhang W, Yao Z, Li H (2008). Nitrogen fixation island and rhizosphere competence traits in the genome of root-associated *Pseudomonas stutzeri* A1501. Proc Natl Acad Sci USA.

[75] Yuan Z, Druzhinina IS, Labbé J, Redman R, Qin Y, Rodriguez R, Zhang C, Tuskan GA, Lin F (2016). Specialized microbiome of a halophyte and its role in helping non-host plants to withstand salinity. Sci Rep.

[76] Zhao S, Zhou N, Zhao ZY, Zhang K, Wu GH, Tian CY (2016). Isolation of endophytic plant growth-promoting bacteria associated with the halophyte *Salicornia europaea* and evaluation of their promoting activity under salt stress. Curr Microbiol.

[77] Zhou N, Zhao S, Tian CY. Effect of halotolerant rhizobacteria isolated from halophytes on the growth of sugar beet (*Beta vulgaris* L.) under salt stress. FEMS Microbiol Lett. 2017;364(11). 10.1093/femsle/fnx091 28460054

[78] Zhu F, Qu L, Hong X, Sun X. Isolation and characterization of a phosphate-solubilizing halophilic bacterium *Kushneria* sp. YCWA18 from Daqiao saltern on the coast of Yellow Sea of China. Evid Based Complement Alternat Med. 2011;615032. 10.1155/2011/615032 PMC3118493 21716683

