# Supplementary material for: Endorhizosphere of indigenous succulent halophytes: a valuable resource of plant growth promoting bacteria
Source: Environ Microbiome. 2023 Mar 18;18:20. doi: 10.1186/s40793-023-00477-x (PMC10024849; doi:10.1186/s40793-023-00477-x)
Supplement: Supplementary file 1 — Additional file 1. Supplementary Information. [file 40793_2023_477_MOESM1_ESM.docx]

***Supplementary Material***

**Title: Endorhizosphere of indigenous succulent halophytes: a valuable resource of plant growth promoting bacteria**

Supplementary table SM1 – distribution of most prominent orders. (k) kingdom; (p) phylum; (c) class; (o) order. *S.m.* – *Suaeda maritima*, *C.a.* – *Camporosma annua*, *S.e.* – *Salicornia europaea*. (/) - rare (<0.1%) or not detected orders. Abundant orders (>1%) are given in bold letters. In the Actinobacteria class, the most prominent orders were Micrococcales and Acidimicrobiales in all three plants, and Streptomycetales and Propionibacteriales in *S. europaea* (Table SM1). Among class of Alphaproteobacteria most prominent orders were Rhizobiales, Rhodobacterales, and Sphingomonadales, among Betaproteobacteria most prominent were Rhodocyclales and Burkholderiales, while in Gammaproteobacteria most abundant were Pseudomonadales, Oceanospirillales, and Xanthomonadales.

| ***S.m.***  **(%)** | ***C.a.***  **(%)** | ***S.e.***  **(%)** | **Taxonomy** |
| --- | --- | --- | --- |
| / | 0.15 | / | k__Archaea;p__Euryarchaeota;c__Halobacteria;o__Halobacteriales |
| / | / | 0.23 | k__Archaea;p__Euryarchaeota;c__Methanomicrobia;o__Methanocellales |
| / | / | 0.23 | k__Archaea;p__Euryarchaeota;c__Methanomicrobia;o__Methanosarcinales |
| 0.15 | / | 0.92 | k__Bacteria;p__Acidobacteria;c__Acidobacteria;o__NA |
| / | / | 0.46 | k__Bacteria;p__Acidobacteria;c__Holophagae;o__NA |
| **3.48** | **1.63** | **3.91** | k__Bacteria;p__Actinobacteria;c__Acidimicrobiia;o__Acidimicrobiales |
| 0.15 | / | 0.23 | k__Bacteria;p__Actinobacteria;c__Actinobacteria;o__Corynebacteriales |
| 0.15 | / | / | k__Bacteria;p__Actinobacteria;c__Actinobacteria;o__Frankiales |
| / | / | **1.15** | k__Bacteria;p__Actinobacteria;c__Actinobacteria;o__Glycomycetales |
| / | 0.45 | 0.46 | k__Bacteria;p__Actinobacteria;c__Actinobacteria;o__Kineosporiales |
| **6.35** | **6.97** | **11.03** | k__Bacteria;p__Actinobacteria;c__Actinobacteria;o__Micrococcales |
| 0.30 | 0.45 | 0.69 | k__Bacteria;p__Actinobacteria;c__Actinobacteria;o__Micromonosporales |
| 0.30 | 0.15 | **3.22** | k__Bacteria;p__Actinobacteria;c__Actinobacteria;o__Propionibacteriales |
| / | / | 0.23 | k__Bacteria;p__Actinobacteria;c__Actinobacteria;o__Pseudonocardiales |
| 0.15 | 0.15 | **4.83** | k__Bacteria;p__Actinobacteria;c__Actinobacteria;o__Streptomycetales |
| / | / | 0.92 | k__Bacteria;p__Actinobacteria;c__Actinobacteria;o__Streptosporangiales |
| **1.21** | 0.59 | **3.68** | k__Bacteria;p__Actinobacteria;c__Nitriliruptoria;o__Euzebiales |
| **1.66** | **1.34** | **1.15** | k__Bacteria;p__Actinobacteria;c__Nitriliruptoria;o__Nitriliruptorales |
| / | / | 0.92 | k__Bacteria;p__Actinobacteria;c__Rubrobacteria;o__Rubrobacterales |
| 0.61 | 0.15 | 0.69 | k__Bacteria;p__Actinobacteria;c__Thermoleophilia;o__Solirubrobacterales |
| **3.03** | **4.15** | **3.22** | k__Bacteria;p__Bacteroidetes;c__Cytophagia;o__Cytophagales |
| 0.30 | 0.15 | / | k__Bacteria;p__Bacteroidetes;c__Cytophagia;o__NA |
| 0.45 | **1.04** | **3.22** | k__Bacteria;p__Bacteroidetes;c__Flavobacteriia;o__Flavobacteriales |
| 0.30 | 0.15 | / | k__Bacteria;p__Bacteroidetes;c__Sphingobacteriia;o__Sphingobacteriales |
| 0.76 | 0.45 | 0.46 | k__Bacteria;p__Chloroflexi;c__Anaerolineae;o__Anaerolineales |
| / | 0.15 | / | k__Bacteria;p__Chloroflexi;c__Ardenticatenia;o__NA |
| / | / | 0.92 | k__Bacteria;p__Chloroflexi;c__NA;o__NA |
| / | / | 0.69 | k__Bacteria;p__Chloroflexi;c__Thermomicrobia;o__NA |
| **2.72** | **1.93** | / | k__Bacteria;p__Cyanobacteria;c__Cyanobacteria;o__NA |
| 0.15 | / | / | k__Bacteria;p__Deinococcus-Thermus;c__Deinococci;o__Deinococcales |
| 0.15 | / | / | k__Bacteria;p__Fibrobacteres;c__Fibrobacteria;o__Fibrobacterales |
| 0.76 | **5.19** | **2.53** | k__Bacteria;p__Firmicutes;c__Bacilli;o__Bacillales |
| **1.21** | 0.89 | 0.46 | k__Bacteria;p__Firmicutes;c__Clostridia;o__Clostridiales |
| / | / | 0.23 | k__Bacteria;p__Gemmatimonadetes;c__Gemmatimonadetes;o__Gemmatimonadales |
| 0.30 | 0.15 | **1.84** | k__Bacteria;p__Gemmatimonadetes;c__Gemmatimonadetes;o__NA |
| 0.91 | 0.30 | 0.46 | k__Bacteria;p__Planctomycetes;c__Planctomycetacia;o__Planctomycetales |
| **1.36** | **1.19** | / | k__Bacteria;p__Proteobacteria;c__Alphaproteobacteria;o__Caulobacterales |
| **12.25** | **9.79** | **5.52** | k__Bacteria;p__Proteobacteria;c__Alphaproteobacteria;o__Rhizobiales |
| **4.08** | **5.34** | **6.44** | k__Bacteria;p__Proteobacteria;c__Alphaproteobacteria;o__Rhodobacterales |
| **1.82** | **1.78** | **2.99** | k__Bacteria;p__Proteobacteria;c__Alphaproteobacteria;o__Rhodospirillales |
| **6.20** | **3.56** | **5.06** | k__Bacteria;p__Proteobacteria;c__Alphaproteobacteria;o__Sphingomonadales |
| **1.82** | **5.34** | **1.15** | k__Bacteria;p__Proteobacteria;c__Betaproteobacteria;o__Burkholderiales |
| 0.15 | 0.45 | / | k__Bacteria;p__Proteobacteria;c__Betaproteobacteria;o__Methylophilales |
| **6.96** | **19.29** | **2.07** | k__Bacteria;p__Proteobacteria;c__Betaproteobacteria;o__Rhodocyclales |
| 0.15 | 0.30 | / | k__Bacteria;p__Proteobacteria;c__Deltaproteobacteria;o__Bdellovibrionales |
| 0.15 | 0.15 | 0.23 | k__Bacteria;p__Proteobacteria;c__Deltaproteobacteria;o__Desulfobacterales |
| 0.15 | / | / | k__Bacteria;p__Proteobacteria;c__Deltaproteobacteria;o__Desulfovibrionales |
| 0.15 | 0.59 | 0.46 | k__Bacteria;p__Proteobacteria;c__Deltaproteobacteria;o__Desulfuromonadales |
| 0.91 | 0.74 | 1.38 | k__Bacteria;p__Proteobacteria;c__Deltaproteobacteria;o__Myxococcales |
| / | 0.30 | / | k__Bacteria;p__Proteobacteria;c__Epsilonproteobacteria;o__Campylobacterales |
| **4.39** | **1.78** | / | k__Bacteria;p__Proteobacteria;c__Gammaproteobacteria;o__Aeromonadales |
| / | 0.15 | 0.23 | k__Bacteria;p__Proteobacteria;c__Gammaproteobacteria;o__Alteromonadales |
| 0.61 | **1.34** | **1.38** | k__Bacteria;p__Proteobacteria;c__Gammaproteobacteria;o__Cellvibrionales |
| **2.57** | 0.59 | 0.92 | k__Bacteria;p__Proteobacteria;c__Gammaproteobacteria;o__Chromatiales |
| 0.76 | 0.15 | / | k__Bacteria;p__Proteobacteria;c__Gammaproteobacteria;o__Enterobacteriales |
| 0.15 | / | / | k__Bacteria;p__Proteobacteria;c__Gammaproteobacteria;o__Legionellales |
| / | 0.30 | / | k__Bacteria;p__Proteobacteria;c__Gammaproteobacteria;o__Methylococcales |
| 0.45 | 0.30 | 0.46 | k__Bacteria;p__Proteobacteria;c__Gammaproteobacteria;o__NA |
| **2.57** | **2.23** | **8.05** | k__Bacteria;p__Proteobacteria;c__Gammaproteobacteria;o__Oceanospirillales |
| **18.76** | **12.17** | **7.36** | k__Bacteria;p__Proteobacteria;c__Gammaproteobacteria;o__Pseudomonadales |
| 0.61 | 0.30 | 0.23 | k__Bacteria;p__Proteobacteria;c__Gammaproteobacteria;o__Thiotrichales |
| / | 0.30 | / | k__Bacteria;p__Proteobacteria;c__Gammaproteobacteria;o__Vibrionales |
| **5.14** | **3.56** | **3.45** | k__Bacteria;p__Proteobacteria;c__Gammaproteobacteria;o__Xanthomonadales |
| 0.61 | / | **2.99** | k__Bacteria;p__Saccharibacteria;c__NA;o__NA |
| 0.30 | 0.30 | / | k__Bacteria;p__Verrucomicrobia;c__Opitutae;o__Opitutales |
| / | / | 0.23 | k__Bacteria;p__Verrucomicrobia;c__Opitutae;o__Puniceicoccales |
| 0.30 | / | 0.23 | k__Bacteria;p__Verrucomicrobia;c__Verrucomicrobiae;o__Verrucomicrobiales |

Supplementary table SM2 – distribution of most prominent families. (k) kingdom; (p) phylum; (c) class; (o) order; (f) family. *S.m.* – *Suaeda maritima*, *C.a.* – *Camporosma annua*, *S.e.* – *Salicornia europaea*. (/) - rare (<0.1%) or not detected families. Abundant families (>1%) are given in bold letters.

| ***S.m.*** | ***C.a.*** | ***S.e.*** | **Taxonomy** |
| --- | --- | --- | --- |
| **(%)** | **(%)** | **(%)** |  |
| **/** | 0.15 | **/** | k__Archaea;p__Euryarchaeota;c__Halobacteria;o__Halobacteriales;f__Halobacteriaceae |
| **/** | **/** | 0.23 | k__Archaea;p__Euryarchaeota;c__Methanomicrobia;o__Methanocellales;f__Methanocellaceae |
| **/** | **/** | 0.23 | k__Archaea;p__Euryarchaeota;c__Methanomicrobia;o__Methanosarcinales;f__Methanosarcinaceae |
| 0.15 | **/** | 0.92 | k__Bacteria;p__Acidobacteria;c__Acidobacteria;o__NA;f__NA |
| **/** | **/** | 0.46 | k__Bacteria;p__Acidobacteria;c__Holophagae;o__NA;f__NA |
| **1.21** | 0.74 | 0.92 | k__Bacteria;p__Actinobacteria;c__Acidimicrobiia;o__Acidimicrobiales;f__Acidimicrobiaceae |
| 0.45 | 0.15 | 0.23 | k__Bacteria;p__Actinobacteria;c__Acidimicrobiia;o__Acidimicrobiales;f__Iamiaceae |
| **1.82** | 0.74 | **2.76** | k__Bacteria;p__Actinobacteria;c__Acidimicrobiia;o__Acidimicrobiales;f__NA |
| 0.15 | **/** | **/** | k__Bacteria;p__Actinobacteria;c__Actinobacteria;o__Corynebacteriales;f__Dietziaceae |
| **/** | **/** | 0.23 | k__Bacteria;p__Actinobacteria;c__Actinobacteria;o__Corynebacteriales;f__Mycobacteriaceae |
| 0.15 | **/** | **/** | k__Bacteria;p__Actinobacteria;c__Actinobacteria;o__Frankiales;f__NA |
| **/** | **/** | **1.15** | k__Bacteria;p__Actinobacteria;c__Actinobacteria;o__Glycomycetales;f__Glycomycetaceae |
| **/** | 0.45 | 0.46 | k__Bacteria;p__Actinobacteria;c__Actinobacteria;o__Kineosporiales;f__Kineosporiaceae |
| **1.82** | **1.78** | **/** | k__Bacteria;p__Actinobacteria;c__Actinobacteria;o__Micrococcales;f__Cellulomonadaceae |
| 0.45 | 0.15 | 0.46 | k__Bacteria;p__Actinobacteria;c__Actinobacteria;o__Micrococcales;f__Demequinaceae |
| **1.06** | **1.04** | 0.23 | k__Bacteria;p__Actinobacteria;c__Actinobacteria;o__Micrococcales;f__Intrasporangiaceae |
| **2.57** | **3.26** | **/** | k__Bacteria;p__Actinobacteria;c__Actinobacteria;o__Micrococcales;f__Jonesiaceae |
| 0.15 | 0.30 | 0.46 | k__Bacteria;p__Actinobacteria;c__Actinobacteria;o__Micrococcales;f__Microbacteriaceae |
| **/** | 0.30 | **1.84** | k__Bacteria;p__Actinobacteria;c__Actinobacteria;o__Micrococcales;f__Micrococcaceae |
| **/** | **/** | **8.05** | k__Bacteria;p__Actinobacteria;c__Actinobacteria;o__Micrococcales;f__Promicromonosporaceae |
| 0.30 | **/** | 0.23 | k__Bacteria;p__Actinobacteria;c__Actinobacteria;o__Micrococcales;f__Sanguibacteraceae |
| 0.30 | 0.45 | 0.69 | k__Bacteria;p__Actinobacteria;c__Actinobacteria;o__Micromonosporales;f__Micromonosporaceae |
| 0.30 | 0.15 | **3.22** | k__Bacteria;p__Actinobacteria;c__Actinobacteria;o__Propionibacteriales;f__Nocardioidaceae |
| **/** | **/** | 0.23 | k__Bacteria;p__Actinobacteria;c__Actinobacteria;o__Pseudonocardiales;f__Pseudonocardiaceae |
| 0.15 | 0.15 | **4.83** | k__Bacteria;p__Actinobacteria;c__Actinobacteria;o__Streptomycetales;f__Streptomycetaceae |
| **/** | **/** | 0.69 | k__Bacteria;p__Actinobacteria;c__Actinobacteria;o__Streptosporangiales;f__Nocardiopsaceae |
| **/** | **/** | 0.23 | k__Bacteria;p__Actinobacteria;c__Actinobacteria;o__Streptosporangiales;f__Thermomonosporaceae |
| **1.21** | 0.59 | **3.68** | k__Bacteria;p__Actinobacteria;c__Nitriliruptoria;o__Euzebyales;f__Euzebyaceae |
| **1.66** | **1.34** | **1.15** | k__Bacteria;p__Actinobacteria;c__Nitriliruptoria;o__Nitriliruptorales;f__Nitriliruptoraceae |
| **/** | **/** | 0.92 | k__Bacteria;p__Actinobacteria;c__Rubrobacteria;o__Rubrobacterales;f__Rubrobacteriaceae |
| 0.30 | 0.15 | 0.69 | k__Bacteria;p__Actinobacteria;c__Thermoleophilia;o__Solirubrobacterales;f__NA |
| 0.15 | **/** | **/** | k__Bacteria;p__Actinobacteria;c__Thermoleophilia;o__Solirubrobacterales;f__Patulibacteraceae |
| 0.76 | 0.74 | 0.46 | k__Bacteria;p__Bacteroidetes;c__Cytophagia;o__Cytophagales;f__Cyclobacteriaceae |
| 0.61 | **1.48** | 0.46 | k__Bacteria;p__Bacteroidetes;c__Cytophagia;o__Cytophagales;f__Cytophagaceae |
| 0.76 | 0.74 | **1.61** | k__Bacteria;p__Bacteroidetes;c__Cytophagia;o__Cytophagales;f__Flammeovirgaceae |
| **1.06** | **1.04** | 0.92 | k__Bacteria;p__Bacteroidetes;c__Cytophagia;o__Cytophagales;f__NA |
| 0.15 | **/** | **/** | k__Bacteria;p__Bacteroidetes;c__Cytophagia;o__NA;f__NA |
| 0.30 | 0.15 | **/** | k__Bacteria;p__Bacteroidetes;c__Cytophagia;o__NA;f__Rhodothermaceae |
| 0.45 | 0.89 | **3.22** | k__Bacteria;p__Bacteroidetes;c__Flavobacteriia;o__Flavobacteriales;f__Flavobacteriaceae |
| 0.15 | **/** | **/** | k__Bacteria;p__Bacteroidetes;c__Sphingobacteriia;o__Sphingobacteriales;f__Chitinophagaceae |
| 0.15 | **/** | **/** | k__Bacteria;p__Bacteroidetes;c__Sphingobacteriia;o__Sphingobacteriales;f__NA |
| 0.76 | 0.45 | 0.46 | k__Bacteria;p__Chloroflexi;c__Anaerolineae;o__Anaerolineales;f__Anaerolineaceae |
| **/** | 0.15 | **/** | k__Bacteria;p__Chloroflexi;c__Ardenticatenia;o__NA;f__NA |
| **/** | **/** | 0.92 | k__Bacteria;p__Chloroflexi;c__NA;o__NA;f__NA |
| **/** | **/** | 0.69 | k__Bacteria;p__Chloroflexi;c__Thermomicrobia;o__NA;f__NA |
| **2.72** | **1.93** | **/** | k__Bacteria;p__Cyanobacteria;c__Cyanobacteria;o__NA;f__FamilyI |
| 0.15 | **/** | **/** | k__Bacteria;p__Deinococcus-Thermus;c__Deinococci;o__Deinococcales;f__Trueperaceae |
| 0.15 | **/** | **/** | k__Bacteria;p__Fibrobacteres;c__Fibrobacteria;o__Fibrobacterales;f__Fibrobacteraceae |
| 0.61 | 0.45 | **1.84** | k__Bacteria;p__Firmicutes;c__Bacilli;o__Bacillales;f__Bacillaceae |
| 0.15 | **3.41** | 0.23 | k__Bacteria;p__Firmicutes;c__Bacilli;o__Bacillales;f__Family XII |
| **/** | 0.15 | 0.23 | k__Bacteria;p__Firmicutes;c__Bacilli;o__Bacillales;f__Paenibacillaceae |
| **/** | **1.19** | 0.46 | k__Bacteria;p__Firmicutes;c__Bacilli;o__Bacillales;f__Planococcaceae |
| 0.15 | **/** | **/** | k__Bacteria;p__Firmicutes;c__Clostridia;o__Clostridiales;f__Family XII |
| **/** | **/** | 0.23 | k__Bacteria;p__Firmicutes;c__Clostridia;o__Clostridiales;f__Gracilibacteraceae |
| 0.61 | 0.30 | **/** | k__Bacteria;p__Firmicutes;c__Clostridia;o__Clostridiales;f__Lachnospiraceae |
| 0.15 | 0.45 | **/** | k__Bacteria;p__Firmicutes;c__Clostridia;o__Clostridiales;f__NA |
| 0.30 | 0.15 | **/** | k__Bacteria;p__Firmicutes;c__Clostridia;o__Clostridiales;f__Peptostreptococcaceae |
| 0.15 | **/** | 0.23 | k__Bacteria;p__Firmicutes;c__Clostridia;o__Clostridiales;f__Ruminococcaceae |
| **/** | **/** | 0.23 | k__Bacteria;p__Gemmatimonadetes;c__Gemmatimonadetes;o__Gemmatimonadales;f__Gemmatimonadaceae |
| 0.30 | 0.15 | **1.84** | k__Bacteria;p__Gemmatimonadetes;c__Gemmatimonadetes;o__NA;f__NA |
| 0.91 | 0.30 | 0.46 | k__Bacteria;p__Planctomycetes;c__Planctomycetacia;o__Planctomycetales;f__Planctomycetaceae |
| **1.21** | **1.04** | **/** | k__Bacteria;p__Proteobacteria;c__Alphaproteobacteria;o__Caulobacterales;f__Caulobacteraceae |
| 0.15 | **/** | **/** | k__Bacteria;p__Proteobacteria;c__Alphaproteobacteria;o__Caulobacterales;f__Hyphomonadaceae |
| 0.45 | 0.15 | **/** | k__Bacteria;p__Proteobacteria;c__Alphaproteobacteria;o__Rhizobiales;f__Bradyrhizobiaceae |
| **4.54** | **2.52** | **1.15** | k__Bacteria;p__Proteobacteria;c__Alphaproteobacteria;o__Rhizobiales;f__Hyphomicrobiaceae |
| 0.15 | 0.15 | **/** | k__Bacteria;p__Proteobacteria;c__Alphaproteobacteria;o__Rhizobiales;f__Methylobacteriaceae |
| **/** | **/** | 0.23 | k__Bacteria;p__Proteobacteria;c__Alphaproteobacteria;o__Rhizobiales;f__Methylocystaceae |
| 0.15 | 0.30 | 0.46 | k__Bacteria;p__Proteobacteria;c__Alphaproteobacteria;o__Rhizobiales;f__NA |
| **1.97** | **1.34** | **1.61** | k__Bacteria;p__Proteobacteria;c__Alphaproteobacteria;o__Rhizobiales;f__Phyllobacteriaceae |
| **4.39** | **4.90** | **1.38** | k__Bacteria;p__Proteobacteria;c__Alphaproteobacteria;o__Rhizobiales;f__Rhizobiaceae |
| 0.76 | 0.45 | 0.46 | k__Bacteria;p__Proteobacteria;c__Alphaproteobacteria;o__Rhizobiales;f__Rhodobiaceae |
| **4.08** | **5.34** | **6.44** | k__Bacteria;p__Proteobacteria;c__Alphaproteobacteria;o__Rhodobacterales;f__Rhodobacteraceae |
| 0.15 | 0.15 | **/** | k__Bacteria;p__Proteobacteria;c__Alphaproteobacteria;o__Rhodospirillales;f__Acetobacteraceae |
| **/** | **/** | **1.61** | k__Bacteria;p__Proteobacteria;c__Alphaproteobacteria;o__Rhodospirillales;f__NA |
| **1.66** | **1.63** | **1.38** | k__Bacteria;p__Proteobacteria;c__Alphaproteobacteria;o__Rhodospirillales;f__Rhodospirillaceae |
| **4.84** | **2.97** | **3.45** | k__Bacteria;p__Proteobacteria;c__Alphaproteobacteria;o__Sphingomonadales;f__Erythrobacteraceae |
| **1.36** | 0.59 | **1.38** | k__Bacteria;p__Proteobacteria;c__Alphaproteobacteria;o__Sphingomonadales;f__Sphingomonadaceae |
| **1.66** | **5.34** | 0.69 | k__Bacteria;p__Proteobacteria;c__Betaproteobacteria;o__Burkholderiales;f__Comamonadaceae |
| 0.15 | **/** | 0.23 | k__Bacteria;p__Proteobacteria;c__Betaproteobacteria;o__Burkholderiales;f__Oxalobacteraceae |
| 0.15 | 0.45 | **/** | k__Bacteria;p__Proteobacteria;c__Betaproteobacteria;o__Methylophilales;f__Methylophilaceae |
| **6.96** | **19.29** | **2.07** | k__Bacteria;p__Proteobacteria;c__Betaproteobacteria;o__Rhodocyclales;f__Rhodocyclaceae |
| 0.15 | 0.15 | **/** | k__Bacteria;p__Proteobacteria;c__Deltaproteobacteria;o__Bdellovibrionales;f__Bacteriovoracaceae |
| **/** | 0.15 | **/** | k__Bacteria;p__Proteobacteria;c__Deltaproteobacteria;o__Bdellovibrionales;f__Bdellovibrionaceae |
| 0.15 | 0.15 | 0.23 | k__Bacteria;p__Proteobacteria;c__Deltaproteobacteria;o__Desulfobacterales;f__Desulfobulbaceae |
| 0.15 | **/** | **/** | k__Bacteria;p__Proteobacteria;c__Deltaproteobacteria;o__Desulfovibrionales;f__Desulfovibrionaceae |
| 0.15 | 0.59 | 0.23 | k__Bacteria;p__Proteobacteria;c__Deltaproteobacteria;o__Desulfuromonadales;f__Desulfuromonadaceae |
| **/** | **/** | 0.23 | k__Bacteria;p__Proteobacteria;c__Deltaproteobacteria;o__Desulfuromonadales;f__NA |
| **/** | **/** | 0.23 | k__Bacteria;p__Proteobacteria;c__Deltaproteobacteria;o__Myxococcales;f__Cystobacteraceae |
| **/** | 0.15 | **/** | k__Bacteria;p__Proteobacteria;c__Deltaproteobacteria;o__Myxococcales;f__Haliangiaceae |
| 0.15 | **/** | **/** | k__Bacteria;p__Proteobacteria;c__Deltaproteobacteria;o__Myxococcales;f__Myxococcaceae |
| 0.15 | 0.15 | 0.46 | k__Bacteria;p__Proteobacteria;c__Deltaproteobacteria;o__Myxococcales;f__NA |
| **/** | 0.15 | **/** | k__Bacteria;p__Proteobacteria;c__Deltaproteobacteria;o__Myxococcales;f__Nannocystaceae |
| 0.61 | 0.45 | 0.46 | k__Bacteria;p__Proteobacteria;c__Deltaproteobacteria;o__Myxococcales;f__Sandaracinaceae |
| **/** | 0.30 | **/** | k__Bacteria;p__Proteobacteria;c__Epsilonproteobacteria;o__Campylobacterales;f__Helicobacteraceae |
| **4.39** | **1.78** | **/** | k__Bacteria;p__Proteobacteria;c__Gammaproteobacteria;o__Aeromonadales;f__Aeromonadaceae |
| **/** | **/** | 0.23 | k__Bacteria;p__Proteobacteria;c__Gammaproteobacteria;o__Alteromonadales;f__Alteromonadaceae |
| **/** | 0.15 | **/** | k__Bacteria;p__Proteobacteria;c__Gammaproteobacteria;o__Alteromonadales;f__Shewanellaceae |
| 0.30 | **1.04** | 0.23 | k__Bacteria;p__Proteobacteria;c__Gammaproteobacteria;o__Cellvibrionales;f__Cellvibrionaceae |
| 0.15 | 0.15 | 0.69 | k__Bacteria;p__Proteobacteria;c__Gammaproteobacteria;o__Cellvibrionales;f__Halieaceae |
| 0.15 | 0.15 | 0.23 | k__Bacteria;p__Proteobacteria;c__Gammaproteobacteria;o__Cellvibrionales;f__Microbulbiferaceae |
| **/** | **/** | 0.23 | k__Bacteria;p__Proteobacteria;c__Gammaproteobacteria;o__Cellvibrionales;f__Porticoccaceae |
| **2.57** | 0.45 | 0.69 | k__Bacteria;p__Proteobacteria;c__Gammaproteobacteria;o__Chromatiales;f__Chromatiaceae |
| **/** | 0.15 | 0.23 | k__Bacteria;p__Proteobacteria;c__Gammaproteobacteria;o__Chromatiales;f__Ectothiorhodospiraceae |
| 0.76 | 0.15 | **/** | k__Bacteria;p__Proteobacteria;c__Gammaproteobacteria;o__Enterobacteriales;f__Enterobacteriaceae |
| **/** | 0.30 | **/** | k__Bacteria;p__Proteobacteria;c__Gammaproteobacteria;o__Methylococcales;f__Methylococcaceae |
| 0.45 | 0.30 | 0.46 | k__Bacteria;p__Proteobacteria;c__Gammaproteobacteria;o__NA;f__NA |
| **1.97** | **1.78** | **7.82** | k__Bacteria;p__Proteobacteria;c__Gammaproteobacteria;o__Oceanospirillales;f__Halomonadaceae |
| 0.61 | 0.45 | **/** | k__Bacteria;p__Proteobacteria;c__Gammaproteobacteria;o__Oceanospirillales;f__Oceanospirillaceae |
| **2.27** | 0.15 | **1.61** | k__Bacteria;p__Proteobacteria;c__Gammaproteobacteria;o__Pseudomonadales;f__Moraxellaceae |
| **16.64** | **12.02** | **5.98** | k__Bacteria;p__Proteobacteria;c__Gammaproteobacteria;o__Pseudomonadales;f__Pseudomonadaceae |
| 0.61 | 0.30 | 0.23 | k__Bacteria;p__Proteobacteria;c__Gammaproteobacteria;o__Thiotrichales;f__Piscirickettsiaceae |
| **/** | 0.30 | **/** | k__Bacteria;p__Proteobacteria;c__Gammaproteobacteria;o__Vibrionales;f__Vibrionaceae |
| 0.15 | 0.15 | 0.92 | k__Bacteria;p__Proteobacteria;c__Gammaproteobacteria;o__Xanthomonadales;f__NA |
| **4.99** | **3.41** | **2.53** | k__Bacteria;p__Proteobacteria;c__Gammaproteobacteria;o__Xanthomonadales;f__Xanthomonadaceae |
| 0.61 | **/** | **2.99** | k__Bacteria;p__Saccharibacteria;c__NA;o__NA;f__NA |
| 0.30 | 0.30 | **/** | k__Bacteria;p__Verrucomicrobia;c__Opitutae;o__Opitutales;f__Opitutaceae |
| **/** | **/** | 0.23 | k__Bacteria;p__Verrucomicrobia;c__Opitutae;o__Puniceicoccales;f__Puniceicoccaceae |
| 0.30 | **/** | 0.23 | k__Bacteria;p__Verrucomicrobia;c__Verrucomicrobiae;o__Verrucomicrobiales;f__Verrucomicrobiaceae |

Supplementary figure SM3 – PcoA plot of measurement of microbial diversity differences between samples. Each dot in the figure represents the whole microbial composition profile on the genus level.


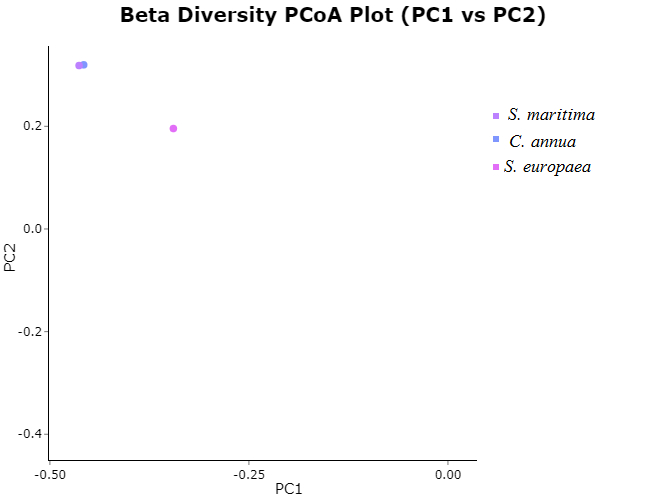


Supplementary table SM4 – distribution of most prominent genera with relative abundance >1% in at least one metagenome. (/) – abundance below 0.1% or genus not detected.

|  | ***S. maritima***  (%) | ***C. annua***  (%) | ***S. europaea***  (%) | **Genus** |
| --- | --- | --- | --- | --- |
| 1 | 1.66 | 0.59 | 2.76 | NA *(Acidimocrobiales)* |
| 2 | / | / | 1.15 | *Glycomyces* |
| 3 | 1.21 | 0.59 | / | *Actinotalea* |
| 4 | 0.61 | 1.19 | / | *Cellulomonas* |
| 5 | 2.57 | 3.26 | / | *Jonesia* |
| 6 | / | / | 5.52 | *Isoptericola* |
| 7 | / | / | 2.53 | *Promicromonospora* |
| 8 | 0.15 | 0.15 | 3.22 | *Nocardioides* |
| 9 | 0.15 | 0.15 | 4.83 | *Streptomyces* |
| 10 | 1.21 | 0.59 | 3.68 | *Euzebya* |
| 11 | 1.66 | 1.34 | 1.15 | *Nitriliruptor* |
| 12 | 1.06 | 1.04 | 0.92 | NA *(Cytophagales)* |
| 13 | / | / | 1.84 | NA *(Flavobacteriaceae)* |
| 14 | 1.66 | 0.74 | / | *Leptolyngbya* |
| 15 | 0.15 | 3.41 | 0.23 | *Exiguobacterium* |
| 16 | 0.30 | 0.15 | 1.84 | NA *(Gemmatimonadetes)* |
| 17 | 1.21 | 0.89 | 1.15 | *Devosia* |
| 18 | 2.57 | 1.34 | / | *Pelagibacterium* |
| 19 | 4.39 | 4.90 | 1.15 | *Rhizobium* |
| 20 | / | / | 2.99 | *Labrenzia* |
| 21 | 2.57 | 3.86 | 2.07 | *Paracoccus* |
| 22 | / | / | 1.38 | NA (*Rhodospirillales*) |
| 23 | 3.33 | 1.63 | 1.15 | *Altererythrobacter* |
| 24 | / | / | 1.38 | *Erythrobacter* |
| 25 | 1.21 | 1.19 | / | *Porphyrobacter* |
| 26 | 1.21 | 4.75 | 0.69 | *Hydrogenophaga* |
| 27 | 6.05 | 17.66 | 2.07 | *Azoarcus* |
| 28 | 4.24 | 1.78 | / | *Oceanisphaera* |
| 29 | 2.57 | 0.45 | 0.69 | *Rheinheimera* |
| 30 | 1.97 | 1.78 | 7.82 | *Halomonas* |
| 31 | 2.27 | 0.15 | / | *Acinetobacter* |
| 32 | / | / | 1.38 | NA*(Moraxellacea)* |
| 33 | 16.64 | 12.02 | 5.98 | *Pseudomonas* |
| 34 | 2.12 | 1.04 | 1.38 | *Luteimonas* |
| 35 | 1.51 | 0.30 | / | *Pseudofulvimonas* |
| 36 | 0.61 | / | 2.99 | NA*(Saccharibacteria)* |
| 37 | 33.13 | 33.09 | 36.09 | Other |

Supplementary table SM5 – The list of OTUs with high identity to database sequences corresponding to species level, present in all three plant endophyte metagenomes. (k) kingdom; (p) phylum; (c) class; (o) order; (f) family; (g) genus; (s) species.

|  | **Taxonomy** |
| --- | --- |
| 1 | k__Bacteria;p__Actinobacteria;c__Acidimicrobiia;o__Acidimicrobiales;f__Acidimicrobiaceae;g__Illumatobacter;s__sp4235 |
| 2 | k__Bacteria;p__Actinobacteria;c__Acidimicrobiia;o__Acidimicrobiales;f__Acidimicrobiaceae;g__Illumatobacter;s__sp4235 |
| 3 | k__Bacteria;p__Actinobacteria;c__Acidimicrobiia;o__Acidimicrobiales;f__NA;g__NA;s__sp4475 |
| 4 | k__Bacteria;p__Actinobacteria;c__Acidimicrobiia;o__Acidimicrobiales;f__NA;g__NA;s__sp4642 |
| 5 | k__Bacteria;p__Actinobacteria;c__Actinobacteria;o__Micrococcales;f__Cellulomonadaceae;g__Cellulomonas;s__oligotrophica |
| 6 | k__Bacteria;p__Actinobacteria;c__Actinobacteria;o__Micrococcales;f__Demequinaceae;g__Demequina;s__aestuarii |
| 7 | k__Bacteria;p__Actinobacteria;c__Actinobacteria;o__Micrococcales;f__Intrasporangiaceae;g__Ornithinimicrobium;s__murale-sp6491 |
| 8 | k__Bacteria;p__Actinobacteria;c__Actinobacteria;o__Micrococcales;f__Sanguibacteraceae;g__Sanguibacter;s__antarcticus |
| 9 | k__Bacteria;p__Actinobacteria;c__Actinobacteria;o__Propionibacteriales;f__Nocardioidaceae;g__Nocardioides;s__sp7689 |
| 10 | k__Bacteria;p__Actinobacteria;c__Actinobacteria;o__Streptomycetales;f__Streptomycetaceae;g__Streptomyces;s__fukangensis |
| 11 | k__Bacteria;p__Actinobacteria;c__Nitriliruptoria;o__Euzebyales;f__Euzebyaceae;g__Euzebya;s__sp10628 |
| 12 | k__Bacteria;p__Actinobacteria;c__Nitriliruptoria;o__Euzebyales;f__Euzebyaceae;g__Euzebya;s__sp10633 |
| 13 | k__Bacteria;p__Actinobacteria;c__Nitriliruptoria;o__Nitriliruptorales;f__Nitriliruptoraceae;g__Nitriliruptor;s__alkaliphilus |
| 14 | k__Bacteria;p__Bacteroidetes;c__Cytophagia;o__Cytophagales;f__Flammeovirgaceae;g__Reichenbachiella;s__sp15668 |
| 15 | k__Bacteria;p__Bacteroidetes;c__Cytophagia;o__Cytophagales;f__NA;g__NA;s__sp15786 |
| 16 | k__Bacteria;p__Chloroflexi;c__Anaerolineae;o__Anaerolineales;f__Anaerolineaceae;g__NA;s__sp20579 |
| 17 | k__Bacteria;p__Firmicutes;c__Bacilli;o__Bacillales;f__Bacillaceae;g__Bacillus;s__aryabhattai-flexus-megaterium |
| 18 | k__Bacteria;p__Firmicutes;c__Bacilli;o__Bacillales;f__Bacillaceae;g__Bacillus;s__aryabhattai-flexus-megaterium |
| 19 | k__Bacteria;p__Proteobacteria;c__Alphaproteobacteria;o__Rhizobiales;f__Phyllobacteriaceae;g__Hoeflea;s__marina |
| 20 | k__Bacteria;p__Proteobacteria;c__Alphaproteobacteria;o__Rhizobiales;f__Phyllobacteriaceae;g__Mesorhizobium;s__sp44061 |
| 21 | k__Bacteria;p__Proteobacteria;c__Alphaproteobacteria;o__Rhizobiales;f__Rhizobiaceae;g__Rhizobium;s__leguminosarum |
| 22 | k__Bacteria;p__Proteobacteria;c__Alphaproteobacteria;o__Rhizobiales;f__Rhizobiaceae;g__Rhizobium;s__rosettiformans-vitis |
| 23 | k__Bacteria;p__Proteobacteria;c__Alphaproteobacteria;o__Rhizobiales;f__Rhizobiaceae;g__Rhizobium;s__rubi |
| 24 | k__Bacteria;p__Proteobacteria;c__Alphaproteobacteria;o__Rhodobacterales;f__Rhodobacteraceae;g__Paracoccus;s__carotinifaciens-marcusii |
| 25 | k__Bacteria;p__Proteobacteria;c__Alphaproteobacteria;o__Rhodobacterales;f__Rhodobacteraceae;g__Paracoccus;s__tibetensis |
| 26 | k__Bacteria;p__Proteobacteria;c__Alphaproteobacteria;o__Sphingomonadales;f__Erythrobacteraceae;g__NA;s__NA |
| 27 | k__Bacteria;p__Proteobacteria;c__Betaproteobacteria;o__Burkholderiales;f__Comamonadaceae;g__Hydrogenophaga;s__bisanensis-pseudoflava |
| 28 | k__Bacteria;p__Proteobacteria;c__Betaproteobacteria;o__Rhodocyclales;f__Rhodocyclaceae;g__Azoarcus;s__sp50297-sp50303 |
| 29 | k__Bacteria;p__Proteobacteria;c__Deltaproteobacteria;o__Myxococcales;f__NA;g__NA;s__sp53453 |
| 30 | k__Bacteria;p__Proteobacteria;c__Gammaproteobacteria;o__Cellvibrionales;f__Microbulbiferaceae;g__Microbulbifer;s__NA |
| 31 | k__Bacteria;p__Proteobacteria;c__Gammaproteobacteria;o__Chromatiales;f__Chromatiaceae;g__Rheinheimera;s__aquimaris |
| 32 | k__Bacteria;p__Proteobacteria;c__Gammaproteobacteria;o__NA;f__NA;g__NA;s__sp56033 |
| 33 | k__Bacteria;p__Proteobacteria;c__Gammaproteobacteria;o__Oceanospirillales;f__Halomonadaceae;g__Halomonas;s__nitritophilus |
| 34 | k__Bacteria;p__Proteobacteria;c__Gammaproteobacteria;o__Oceanospirillales;f__Halomonadaceae;g__Halomonas;s__songnenensis |
| 35 | k__Bacteria;p__Proteobacteria;c__Gammaproteobacteria;o__Pseudomonadales;f__Pseudomonadaceae;g__Pseudomonas;s__NA |
| 36 | k__Bacteria;p__Proteobacteria;c__Gammaproteobacteria;o__Pseudomonadales;f__Pseudomonadaceae;g__Pseudomonas;s__anguilliseptica-guineae-peli |
| 37 | k__Bacteria;p__Proteobacteria;c__Gammaproteobacteria;o__Pseudomonadales;f__Pseudomonadaceae;g__Pseudomonas;s__mendocina |
| 38 | k__Bacteria;p__Proteobacteria;c__Gammaproteobacteria;o__Pseudomonadales;f__Pseudomonadaceae;g__Pseudomonas;s__pseudoalcaligenes |
| 39 | k__Bacteria;p__Proteobacteria;c__Gammaproteobacteria;o__Pseudomonadales;f__Pseudomonadaceae;g__Pseudomonas;s__stutzeri |
| 40 | k__Bacteria;p__Proteobacteria;c__Gammaproteobacteria;o__Pseudomonadales;f__Pseudomonadaceae;g__Pseudomonas;s__stutzeri-xanthomarina-zhaodongensis |
| 41 | k__Bacteria;p__Proteobacteria;c__Gammaproteobacteria;o__Xanthomonadales;f__Xanthomonadaceae;g__Arenimonas;s__donghaensis |
| 42 | k__Bacteria;p__Proteobacteria;c__Gammaproteobacteria;o__Xanthomonadales;f__Xanthomonadaceae;g__Arenimonas;s__metalli |
| 43 | k__Bacteria;p__Proteobacteria;c__Gammaproteobacteria;o__Xanthomonadales;f__Xanthomonadaceae;g__Luteimonas;s__aestuarii |
